# NOD2 reduces the chemoresistance of melanoma by inhibiting the TYMS/PLK1 signaling axis

**DOI:** 10.1038/s41419-024-07104-8

**Published:** 2024-10-01

**Authors:** Fang Yun, Na Wu, Xiaojia Yi, Xuedan Zhang, Yu Feng, Qinxuan Ni, Yanlong Gai, Enjiang Li, Zhe Yang, Qiao Zhang, Buqing Sai, Yingmin Kuang, Yuechun Zhu

**Affiliations:** 1 Department of Biochemistry and Molecular Biology, School of Basic Medical Sciences, Kunming Medical University, Kunming, China; 2 Department of Pathology, The Second Affiliated Hospital of Kunming Medical University, Kunming, China; 3 Department of Pathology, The First Affiliated Hospital of Kunming Medical University, Kunming, China; 4 Department of Organ Transplantation, The First Affiliated Hospital of Kunming Medical University, Kunming, China

**Keywords:** Melanoma, Mechanisms of disease, Ubiquitylation, Transcriptomics

## Abstract

Nucleotide-binding oligomerization domain 2 (NOD2) is an immune sensor crucial for eliciting the innate immune responses. Nevertheless, discrepancies exist regarding the effect of NOD2 on different types of cancer. This study aimed to investigate these function of NOD2 in melanoma and its underlying mechanisms. We have validated the tumor suppressor effect of NOD2 in melanoma. NOD2 inhibited the proliferation of melanoma cells, hindering their migration and invasion while promoting the onset of apoptosis. Our study showed that NOD2 expression is closely related to pyrimidine and folate metabolism. NOD2 inhibits thymidylate synthase (TYMS) expression by promoting K48-type ubiquitination modification of TYMS, thereby decreasing the resistance of melanoma cells to 5-fluorouracil (5-FU) and capecitabine (CAP). TYMS was identified to form a complex with Polo-like Kinase 1 (PLK1) and activate the PLK1 signaling pathway. Furthermore, we revealed that the combination of the PLK1 inhibitor volasertib (BI6727) with 5-FU or CAP had a synergistic effect repressing the proliferation, migration, and autophagy of melanoma cells. Overall, our research highlights the protective role of NOD2 in melanoma and suggests that targeting NOD2 and the TYMS/PLK1 signaling axis is a high-profile therapy that could be a prospect for melanoma treatment.

## Introduction

Melanoma is one of the ten most dangerous tumors, with rapid progression, easy metastasis, and a poor prognosis. It is expected that by 2024, there will be 100,640 new cases of cutaneous melanoma and 8290 deaths worldwide [1]. Although BRAF/MEK inhibitors and anti-PD-1 immunotherapies have improved efficacy to some extent, toxicity and drug resistance remain unresolved effectively [2–4]. Several studies have shown that combination therapy effectively reduces drug resistance and improves anti-tumor efficacy [5–7]. Therefore, exploring the molecular mechanisms of melanoma and the impact of combination therapy would help develop new therapeutic strategies.

Nucleotide-binding oligomerization domain 2 (NOD2) is an intracellular pattern recognition receptor in the immune response [8]. Reported studies have clarified the critical function of NOD2 in controlling inflammatory diseases and invasion of host microorganisms, like Crohn’s disease [9], colitis [10], Blau syndrome [11], and meningitis [12]. Recent investigations have pointed out that aberrant NOD2 expression is tied to cancer progression. NOD2 inhibited the proliferation of esophageal adenocarcinoma cells via autophagy [13], whereas its deficiency promoted colorectal tumorigenesis [14]. Upregulation of NOD2 enhanced the proliferation, invasion, and migration of cervical squamous cell carcinoma [15]. NOD2 also promoted hepatocellular carcinoma development through DNA damage-induced autophagy mechanism [16]. These findings reveal that NOD2 plays a complex role in cancer and that specific manifestations may exist across different tissues. In addition, NOD2 agonists or antagonists exert adjuvant effects in immune checkpoint inhibitor therapy. Combining NOD2 agonists with PD-1/PD-L1 immune checkpoint inhibitors for Alzheimer’s disease has revealed their synergistic impact [17]. Covalently coupled NOD2 and toll-like receptor 7 (TLR7) agonists showed potent immunostimulatory activity on immune cells [18]. Coupling of NOD2 antagonist and paclitaxel (PTX) enhanced the growth inhibitory effect of PTX on Lewis lung carcinoma (LLC)-loaded mice [19]. Although NOD2 exhibits biological functions and therapeutic potential in various diseases, its specific role and mechanism in melanoma require further investigation.

Thymidylate synthase (TYMS) has been spotlighted as an essential target for tumor chemotherapeutic agents [20–22]. TYMS maintains the stability of the thymidine-5-prime monophosphate (dTMP) pool, a key dTMP in DNA replication and repair, by catalyzing the methylation of deoxyuridylate to deoxythymidylate using N5, N10-methylenetetrahydrofolate (methylene-THF) as a cofactor [23]. In addition, TYMS is the site of action of chemotherapeutic agents such as 5-fluorouracil (5-FU), 5-fluoro-2-prime-deoxyuridine (FdUMP) and certain folate analogs [24–26]. Thus, TYMS is believed to play a central part in cancer development. Initial studies have indicated that reducing TYMS expression may increase sensitivity to 5-FU chemotherapy in colorectal cancer patients [27, 28]. Conversely, increased expression of TYMS in hepatocellular carcinoma cells and patient samples was associated with hepatocellular carcinoma progression and resistance to 5-FU [29]. Furthermore, the in vivo decrease of TYMS expression lowered tumor occurrence, slowed tumor progression, and extended survival time in mice [30]. However, the reasons for the dysregulation of TYMS in melanoma and its underlying mechanisms remain unclear.

In this work, we observed that NOD2 expression was decreased in melanoma cells. By upregulating NOD2 expression, we successfully inhibited melanoma growth and confirmed that NOD2 diminished melanoma ability to resist chemotherapeutic drugs by reducing TYMS level and activity. Further studies revealed that NOD2 affects the proteasomal degradation pathway of TYMS, subsequently increasing the K48-linked ubiquitination and reducing the K63-linked ubiquitination of TYMS, thereby downregulating TYMS expression. Altered TYMS expression and activity affect Polo-like Kinase 1 (PLK1) expression and activity, and this regulatory relationship occurs through a direct binding interaction of TYMS with PLK1. PLK1 is a critical cell cycle regulatory protein of the serine/threonine kinase family, closely related to melanoma development and growth [31, 32]. NOD2 suppresses melanoma development by inhibiting the TYMS/PLK1 signaling axis. The results of this study offer clues that unravel the mechanisms of melanoma progression and chemoresistance. Also, the significance of implementing NOD2 and the TYMS/PLK1 signaling axis was evaluated as targets for therapeutic intervention in melanoma.

## Materials and methods

### Cell culture and transfection

Human melanoma cell lines A875, A375, SK-MEL-28, and SK-MEL-110 were obtained from the Cell Resource Center, Institute of Basic Medical Sciences, Chinese Academy of Medical Sciences. HEM is a normal human epidermal melanocyte obtained from the Cell Bank of the Chinese Academy of Sciences. All cell lines were cultured in high-glucose DMEM medium supplemented with 10% fetal bovine serum at 37 °C with 5% CO2. Using NOD2 (sh-NOD2-1, 5′-GGGCAAGACTTCCAGGAATTT-3′; sh-NOD2-2, 5′- GTGCTTCTTTGCCGCGTTCTA-3′; sh-NOD2-3, 5′-GGACTACAACTCTGTGGGTGA-3′) and its control (CON313) and NOD2 overexpression and its control (CON335) lentiviruses infected A875 and SK-MEL-110 cells. These viruses were obtained from GeneChem Co., Ltd. (Shanghai, China). After 48 h of transfection, the cell transfection efficiency was observed under a fluorescence microscope, and the cells were continuously screened by puromycin (Solarbio, #P8230) pressurization until the infection rate reached more than 95%. Real-time PCR and Western blot assessed the effect of gene transfection.

### Real-time PCR

TRIzol reagent (Takara, #9109) was used to extract total RNA from cells and tumor tissues. Synthesize cDNA by reverse transcription of mRNA following the instructions of the Reverse Transcription Kit (Thermo Scientific, #K1622). cDNA was analyzed by Real-time PCR using SYBR Green Master mix (Roche, #04913914001); U6 served as an internal reference gene. The specific primer sequences are detailed in Supplementary Table S1.

### Western blot

Cells and tissues can be effectively lysed using a RIPA lysis buffer (Solarbio, #R0020) containing protease and phosphatase (Solarbio, #P0100) inhibitors. Proteins were quantified by BCA, separated via SDS-PAGE gel electrophoresis, and transferred onto PVDF membranes (Millipore, #IPVH00010), sealed with 5% skimmed milk or 5% BSA (Bio Froxx, #4240GR500) for two hours then incubated overnight with TBST (Solarbio, #T1085) diluted primary antibody according to the instructions at 4 °C. The next day, the secondary antibody of the same genus was used and incubated at room temperature for two hours. ELC chemiluminescence was performed to observe the target bands, and ImageJ software was used to scan the gray values. The primary antibody and secondary antibody are shown in Supplementary Table S2.

### Cell proliferation assay (MTS, colony formation, and EdU staining)

To determine cell viability by MTS assay, cells with NOD2 overexpression and knockdown of A875 and SK-MEL-110 were cultured in 96-well plates (1000 cells/well). MTS (Promega, #CTB169) working solution formulated according to 5:1 (DMEM: MTS) was added at different time points. The absorbance value was detected at 490 nm after one hour of dark culture.

Cells were inoculated into six-well plates (800 cells/well), and fresh medium was replaced every three days. Discontinue the culture when cell colony formation was observed. Cells were washed with PBS, fixed in paraformaldehyde, stained with 0.5% crystal violet for 20 min each, and photographed for counting.

In the EdU incorporation determination, cells were first grown in 24-well plates (3 × 10^4^ cells/well), and the next day, an EdU Kit (Beyotime, # C0078S) was used to stain the cells. Hoechst 33342 was utilized for nuclear staining. Collect fluorescence images of EdU (excitation 590 nm, emission 617 nm) and Hoechst (excitation 350 nm, emission 461 nm) under a 20× objective using a fluorescence microscope (Leica, # DM4B). Randomly select three fields of view and use ImageJ software to evaluate the proportion of EdU-positive cells to the total number of cells in the collected images.

### IC50 assay

Cells were inoculated in 96-well plates at 5000 cells/well density. The next day, cells were exposed to various fluorouracil (5-FU) (MCE, #HY-90006) and capecitabine (CAP) (MCE, #HY-B0016) treatment concentrations. The IC50 was calculated by detecting the absorbance values at 490 nm obtained in the MTS assay described above after 48-h exposure.

### Wound healing assay

Cells were inoculated into a six-well plate (1 × 10^6^ cells/well) and incubated to reach confluence after overnight incubation with serum-free DMEM. Further, treated with 10 μg/mL Mitomycin C (MCE, # HY-13316) for 2 h, the cells were scratched with the tip of a 200-uL pipette, washed using PBS, and cultured with serum-free DMEM. Images were taken under an inverted microscope (4× objective) at 0 and 24 h after wounding. Calculate the wound closure area as follows: wound closure area (fold) = (initial wound area - unhealed wound area 24 h after scratching)/initial wound area.

### Transwell migration and invasion assays

Cells treated with 10 μg/mL Mitomycin C (MCE, # HY-13316) for 2 h were digested with trypsin, and concentration (1000 cells/100 ul) was adjusted to be suspended in DMEM. When performing Transwell invasion experiments, 40 µl of matrix gel (BD, #356234) diluted 1:3 (matrix gel: DMEM) was applied to the upper chamber of the Transwell chamber 2 h in advance, then, cultured the cell suspension (Transwell migration: 100 µl, Transwell invasion: 200 µl) into the top chamber of Transwell chamber (Corning, #3524). Inject 600 uL of medium containing 10% fetal bovine serum into the lower chamber of the Transwell, and cells that had migrated and invaded the bottom of the Transwell were removed after 24 or 48 h. Finally, the migrating and invading cells are fixed and stained with crystal violet, then observed under a microscope (40× objective). Choose random fields of view to take pictures and count.

### Flow cytometry

Cell cycle assays are performed by inoculating cells into a 6-well plate, digesting, and centrifuging. Then, they were slowly added dropwise to 75% pre-cooled ethanol and fixed at 4 °C for 24 h. Cells were collected by centrifugation the next day, stained with a cycle kit (4 A Biotech, #FXP0211), and subjected to flow cytometry detection (BD, BD FACSCelesta^TM^ flow cytometry) and analyzed using FlowJo software.

In apoptosis analysis, cells were inoculated in 6-well plates and treated for apoptosis induction. After staining with Annexin V, 633/PI Staining Kit (Dojindo, #AD11) according to the manufacturer’s instructions, assessed apoptic content through flow cytometry (BD, BD FACSCelesta^TM^ flow cytometer) and analyzed using FlowJo software.

### Co-immunoprecipitation (CO-IP)

The cells were lysed for 30 min on a shaker at 4 °C using weak RIPA lysis buffer (Beyotime, #P0013D), adding protease and protease phosphatase (Solarbio, #P0100) inhibitors. Following centrifugation, the supernatant was divided into three parts. The input tube was frozen at minus 20 °C, and the corresponding primary antibody was mixed in the IgG and IP tubes. After incubation on a shaking table at low temperatures for 8 h, protein A/G-Agarose beads (Roche, #11243233001) were added and gently shaken at 4 °C overnight. After centrifugation, the liquid above the sediment was removed, and the sample was subsequently rinsed thrice with PBS. Finally, PBS and protein loading buffer were added, followed by boiling and eluting the combined protein. Western blot analysis was performed using SDS-PAGE.

### Cellular immunofluorescence (IF)

After inoculating the cells in a slide culture, they were removed and fixed in 4% paraformaldehyde for 15 min, followed by exposure to 0.3% Triton X-100 for 20 min. Next, blocking with BSA (Bio Froxx, #4240GR500) for 2 h, followed by the addition of the primary antibody for overnight incubation at 4 °C. Samples with a fluorescent secondary antibody were incubated in the dark for one hour, and the slide was sealed with an anti-fluorescence quencher containing DAPI (Sigma, #F6057). Fluorescent images of TYMS (Alexa Fluor 594: red), PLK1 (Alexa Fluor 488: green), and nucleus (DAPI: blue) were collected using a confocal microscope (OLYMPUS, #SpinSR10) under a 100× oil immersion objective. The following fluorescent secondary antibodies were utilized: 594 (Proteintech, #SA00013-4) and 488 (Proteintech, #SA00013-1).

### GST Pull-down assay

The fusion gens of GST-TYMS and myc-PLK1 were separately cloned into the expression vectors pGEX-4T-1 and pET28a (+), then expressed individually in E. coli TOP10. The expression vectors and E. coli TOP10 were purchased from Wuhan Jinkai Rui Biological. For the in vitro pull-down assay, performed according to the illustrated procedure (Fitgene, #FI88807), after the bacteria were broken by ultrasonication in lysis buffer, the protein samples of 2 mg of GST (control) or GST-TYMS (experimental) were incubated with 50 μl of glutathione agarose resin for 5 h at 4 °C. The samples were washed with a rinse buffer three times, and then 2 mg of myc-PLK1 protein was mixed into each control and experimental group and incubated overnight at 4 °C. Then, the samples from both groups were centrifuged, washed three times with rinse buffer, and eluted by adding elution buffer for 15 min. Add protein loading buffer to the eluted proteins and heat at 95 °C for 10 min. Finally, the samples were analyzed by immunoblotting.

### TYMS activity assay

The total protein was extracted with RIPA lysis buffer, and the concentration was determined using the BCA method. Following guidelines provided by the manufacturer, TYMS activity was assayed using the TYMS Activity ELISA Kit (MEIMIAN, #MM-0328H1).

### Hematoxylin-eosin (HE) staining and immunohistochemistry (IHC) analysis

Animal tumors were fixed in 4% paraformaldehyde, embedded in paraffin, cut into 5-μm sections, and stored after baking at 65 °C for three hours. HE staining was performed per the manufacturer’s established protocol (Solarbio, #G1120).

For IHC, antigen repair was performed under high pressure using citrate buffer. The endogenous peroxidase activity was inactivated with 3% H2O2, and 5% BSA was blocked, followed by the addition of the primary antibody and overnight incubation at 4 °C. Staining Sections using the DAB Substrate Kit (Biosharp, #BL732A) and hematoxylin staining solution (Biosharp, #517-28-2). Sections were dehydrated again, cleared by a gradient of ethanol and xylene, sealed with neutral glue, and photographed under the microscope (Teksqray, #SQS-1000).

### Bioinformatics analysis

#### Data mining

Mining was conducted for data normalization, and computational analysis was performed of the NOD2 gene expression values obtained from the Gene Expression Omnibus (GEO) database (https://www.ncbi.nlm.nih.gov/geo) for the mRNA expression dataset GSE15605, as well as for normal and primary melanoma tissues and metastatic melanoma tissues in the TCGA database (https://portal.gdc.cancer.gov/). The GEPIA database (http://gepia2.cancer-pku.cn/) was used for the differential analysis of the NOD2, TYMS, and PLK1 genes in melanoma and normal tissues, as well as the correlation between TYMS and PLK1 in melanoma patients. The UALCAN database (https://ualcan.path.uab.edu/) was used for survival prognosis analysis of NOD2 expression and melanoma patients.

For transcriptome sequencing (RNA-seq), SK-MEL-110 cells with NOD2 overexpression and control were collected for RNA-seq by Zhongke New Life in Shanghai, China. After analyzing the high-throughput sequencing data, we identified differentially expressed genes (DEGs) based on the screening criteria: | log2FC | > 1 and Padj < 0.05. Subsequently, Gene Ontology (GO) and Kyoto Encyclopedia of Genes and Genomes (KEGG) analyses were performed on these DEGs to reveal their biological significance.

### Animal models

Six-week-old female BALB/c nude mice were obtained from the Department of Laboratory Animals, Kunming Medical University, and randomly divided into groups. For the no administration of chemotherapeutic drugs treatment group, 1 × 10^7^ of NOD2 overexpression and knockdown A875 cells and their respective control cells were implanted subcutaneously on both sides of nude mice. For the group treated with chemotherapeutic drugs, 1 × 10^7^ A875 cells were implanted into the axilla of nude mice. After two weeks, the nude mice were randomly divided and injected intraperitoneally with saline or chemotherapeutic drugs 5-FU (25 mg/kg) and BI6727 (10 mg/kg) every three days. The diameter of the tumors was measured using vernier calipers every five days during the treatment period. The tumor volume was calculated using the formula: volume = 1/2 × long diameter × wide diameter × wide diameter. In the final stage of the tumor experiment, we euthanized the nude mice and removed tumors for subsequent experiments.

### Statistical analysis

Data were analyzed using GraphPad Prism 2 software. All data were shown as means ± standard deviation (SD) of at least three independent experiments. Before conducting independent samples *t*-tests and one-way analysis of variance (ANOVA), the homogeneity of variance among the groups was assessed. Independent-sample *t*-tests were used to compare differences between two specific groups, and ANOVA was employed to compare differences among multiple groups. *P* < 0.05 was considered to indicate statistically significant differences in the results.

## Results

### NOD2 expression is downregulated in human melanoma, and low expression is associated with poor prognosis for melanoma patients

**Fig. 1:**
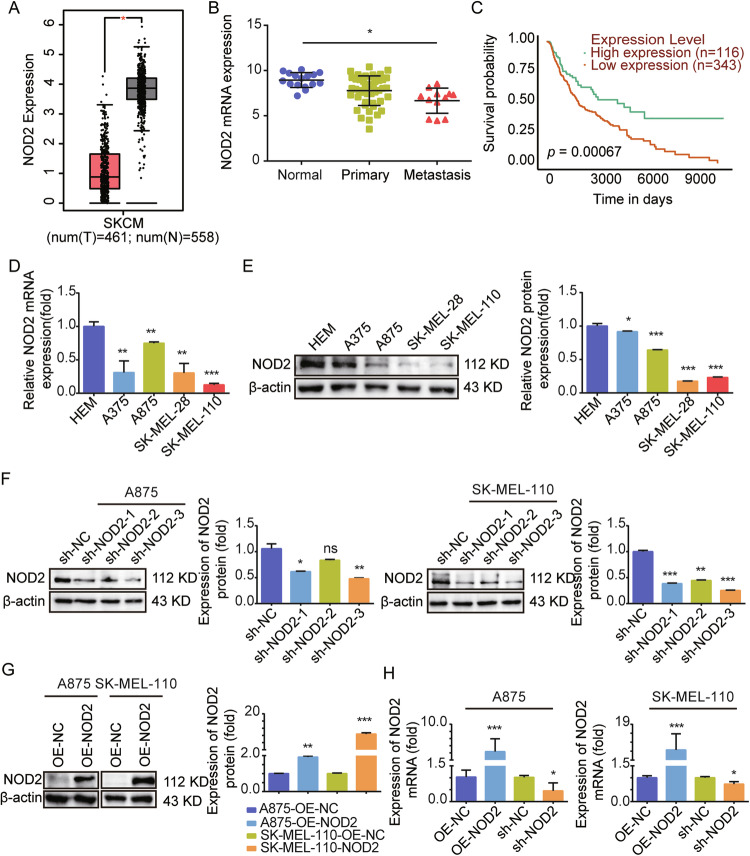
NOD2 is lowly expressed in melanoma and suggests a poor prognosis for melanoma patients. **A** GEPIA database analysis of NOD2 mRNA expression levels in normal and melanoma tissues. **B** TCGA database analysis of NOD2 mRNA expression levels in normal, primary, and metastatic melanoma tissues. **C** Correlation between NOD2 expression in the UALCAN database and prognosis of melanoma patients. **D**, **E** mRNA of NOD2 in human melanocytes HEM and melanoma cells A375, A875, SK-MEL-28, and SK-MEL-110 was detected by real-time PCR (**D**), and the protein expression level was detected by Western blot (**E**). **F**–**H** Construction of stably transfected cells with NOD2 knockdown and overexpression in A875 and SK-MEL-110. Protein expression levels were detected by Western blot (**F**, **G**), and mRNA expression levels were detected by real-time PCR (**H**). Data are expressed as the mean ± SD. Student’s *t*-test and one-way ANOVA were used to compare the differences. *P < 0.05, **P < 0.01, ***P < 0.001. *ns* not significant.

The GEPIA and TCGA databases were used to analyze the role of NOD2 in the development of melanoma, and it was found that the expression of NOD2 was significantly downregulated in melanoma compared to normal tissue, especially in metastatic melanoma patients with lower expression levels (Fig. 1A, B). Moreover, low expression of NOD2 predicts a poor prognosis in melanoma patients (Fig. 1C). Then, we examined NOD2 expression in Human Epidermal Melanocytes (HEM) and four melanoma cell lines. The experimental results revealed a significant decrease in the mRNA expression (Fig. 1D) and protein expression (Fig. 1E) of NOD2 in A375, A875, SK-MEL-28, and SK-MEL-110 cell lines. To further explore the NOD2 function, we successfully constructed stably transfected cell lines with NOD2 knockdown and overexpression in A875 and SK-MEL-110 cells (Fig. 1F–H), with the sh-NOD2-3 sequence having the best knockdown efficiency. Therefore, we used this knockdown sequence cell line in our subsequent experiments and named it sh-NOD2.

### NOD2 inhibits the proliferation of human melanoma cells in vitro

**Fig. 2:**
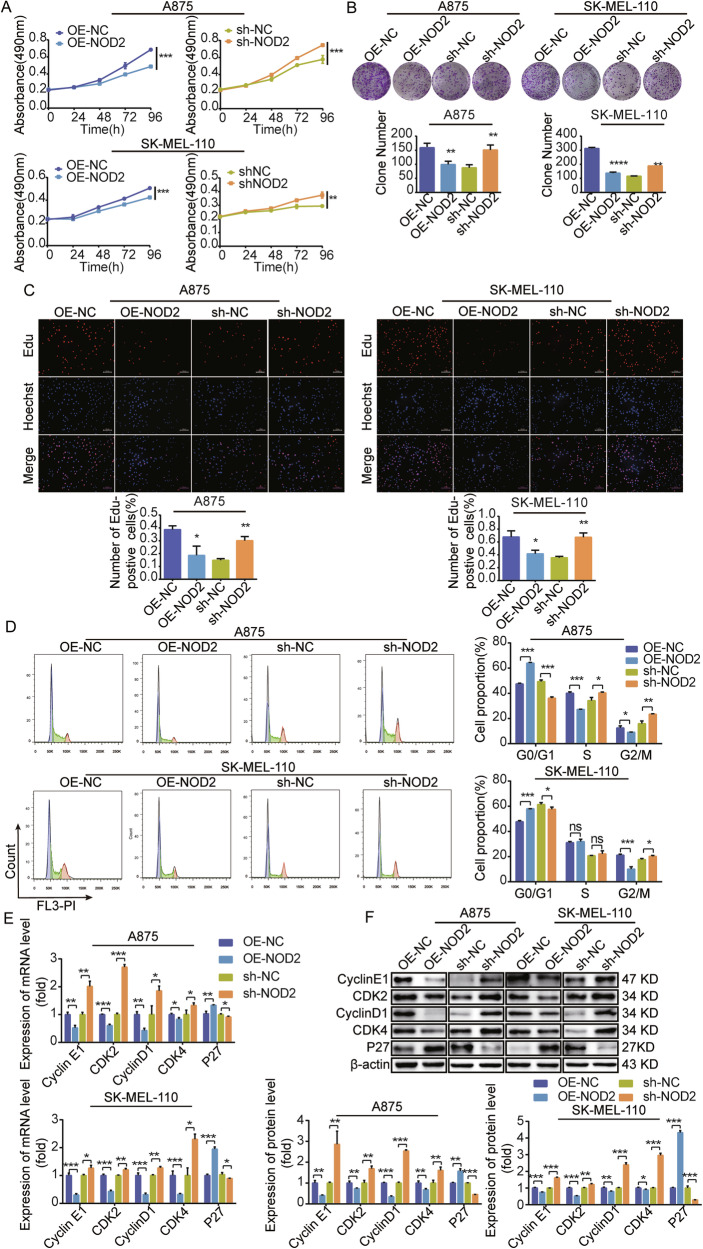
NOD2 inhibits the proliferation of melanoma cells. **A**–**F** Demonstrate the impact of NOD2 overexpression and knockdown on proliferation, colony formation ability, DNA synthesis rate, cell cycle distribution, and expression of mRNA and protein-related cyclins in A875 and SK-MEL-110 cells as determined by different experimental methods. These experimental methods included MTS assay for cell proliferation rate (**A**), colony formation assay for colony size (**B**), and EdU staining for DNA synthesis rate (**C**) (scale bar = 75 μm). The distribution of the cell cycle phase was detected by flow cytometry (**D**). Cyclin expression levels were detected using real-time PCR (**E**) and Western blot (**F**). Data are expressed as the mean ± SD. Student’s *t*-test and one-way ANOVA were used to compare the differences. *P < 0.05, **P < 0.01, ***P < 0.001. *ns* not significant.

We verified the anti-tumor effects of NOD2 in melanoma cells. Overexpression of NOD2 in melanoma inhibited cell proliferative viability, colony-forming ability, and DNA synthesis rate, whereas the opposite effect was observed with NOD2 knockdown (Fig. 2A–C).

We further explored the mechanism of NOD2 role in melanoma cell proliferation. Flow cytometry analysis revealed that NOD2 overexpression in A875 cells led to an increase in G0/G1-phase cells and a decrease in S-phase and G2/M-phase cells. Meanwhile, in SK-MEL-110 cells, NOD2 overexpression also caused an increase in G0/G1-phase cells and decreased G2/M-phase cells, while the number of S-phase cells remained unchanged (Fig. 2D). The results of real-time PCR and Western blot for mRNA (Fig. 2E) and protein (Fig. 2F) expression levels of cell cycle-related factors showed that in melanoma cells with NOD2 overexpression, Cyclin E1/D1 and CDK2/4 expression levels were downregulated while promoting P27 expression. There is an opposite trend in NOD2 knockdown melanoma cells. All of the data obtained further highlight the critical role of NOD2 in inhibiting the proliferation of human melanoma cells.

### NOD2 promotes the apoptosis of melanoma cells

**Fig. 3:**
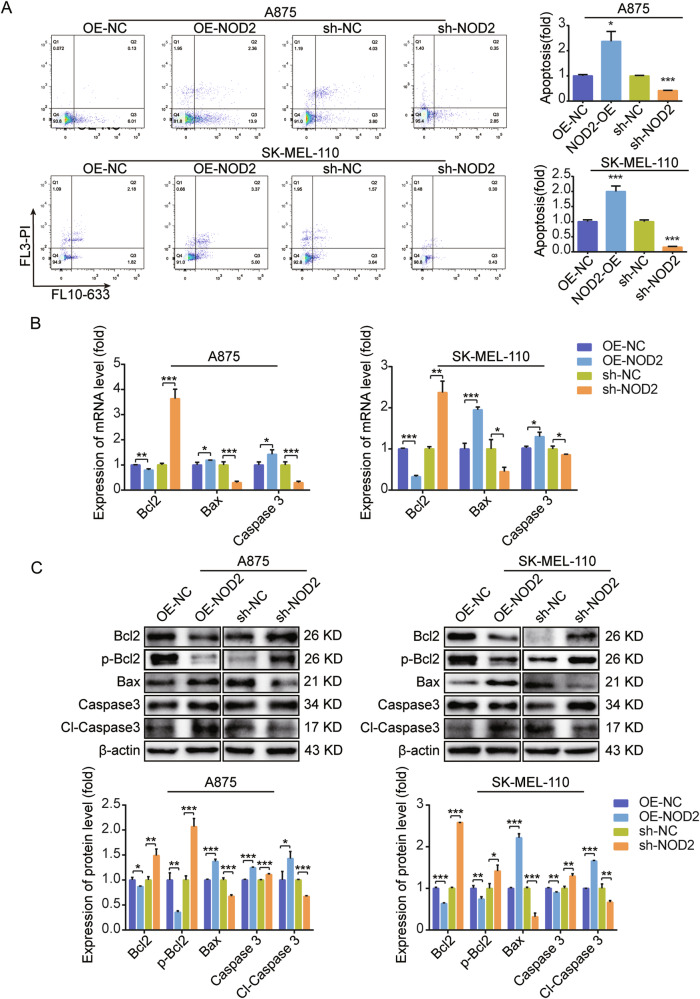
NOD2 promotes apoptosis in human melanoma cells. **A** Apoptosis after overexpression and knockdown of NOD2 in A875 and SK-MEL-110 cells was determined by flow cytometry. **B**, **C** Real-time PCR (**B**) and Western blot (**C**) assays were used to determine the mRNA and protein expression of the apoptotic and anti-apoptotic protein after NOD2 overexpression and knockdown in A875 and SK-MEL-110 cells. Data are expressed as the mean ± SD. Student’s *t*-test and one-way ANOVA were used to compare the differences. *P < 0.05, **P < 0.01, ***P < 0.001.

In investigating the effect of NOD2 on apoptosis in melanoma cells, flow cytometry assays demonstrated that NOD2 overexpression increased the apoptosis rate, while NOD2 knockdown decreased it (Fig. 3A). Further analysis revealed a significant increase in the levels of pro-apoptotic factors Bax and Caspase 3, along with the activated form of Caspase 3 (Cl-Caspase 3), following NOD2 overexpression. Conversely, the anti-apoptotic protein Bcl2 and its activated form p-Bcl2 levels were reduced. NOD2 knockdown promotes apoptosis in melanoma cells (Fig. 3B, C).

### NOD2 inhibits the migration and invasion of human melanoma cells

**Fig. 4:**
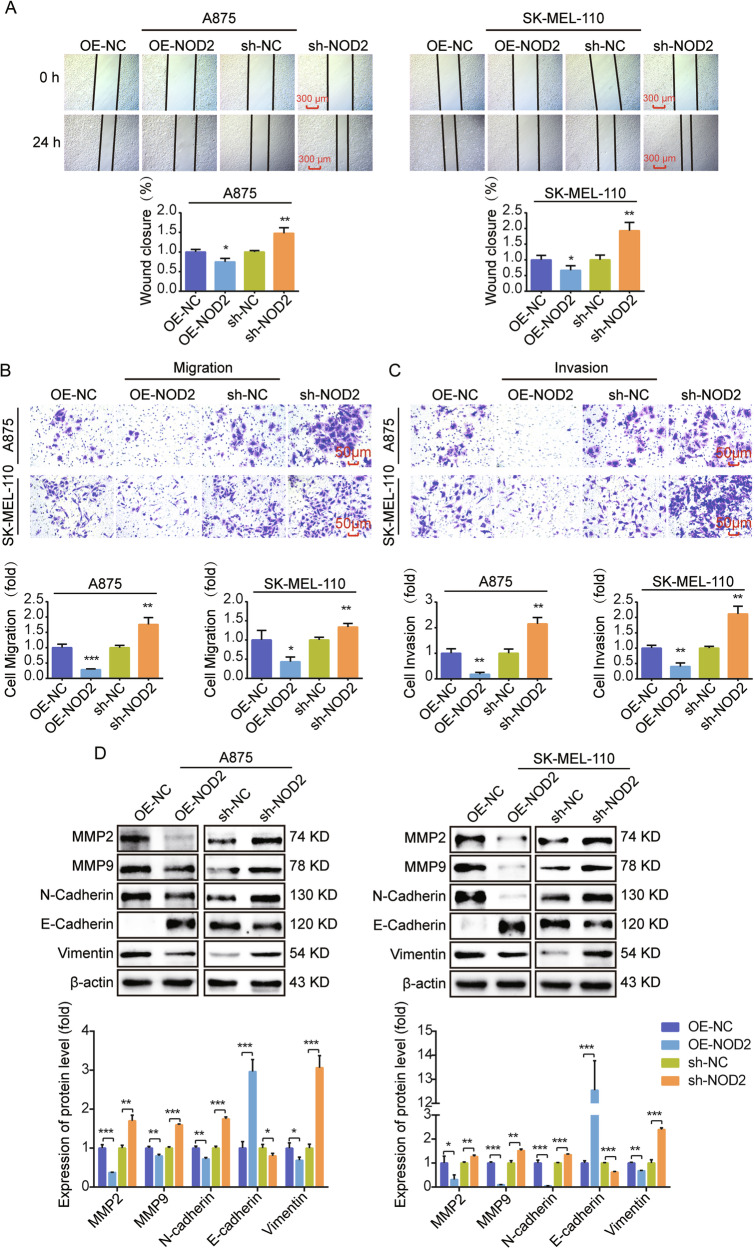
NOD2 inhibits melanoma cell migration and invasion. **A** Scratch assay was used to determine wound healing at 0 h and 24 h after NOD2 overexpression and knockdown in A875 and SK-MEL-110 cells. Scale bar = 300 μm. **B**, **C** Transwell migration assay was used to assess the migration ability of cells, and an invasive assay was used to evaluate the invasive ability of cells. A875 and SK-MEL-110 cells were divided into NOD2 overexpression and knockdown groups, and the number of cells in the lower section was tallied at 24 h. Scale bar = 50 μm. **D** Western blot was used to determine the expression level of EMT protein in NOD2 overexpression and knockdown cells. Data are expressed as the mean ± SD. Student’s *t*-test and one-way ANOVA were used to compare the differences. *P < 0.05, **P < 0.01, ***P < 0.001.

We studied the influence of NOD2 on the migration and invasion ability in human melanoma cells. Scratch and Transwell migration assays were employed to assess cell migration ability, and a Transwell invasion assay was used to evaluate cell invasion ability. The findings indicated that NOD2 overexpression suppressed melanoma cell migration (Fig. 4A, B) and invasion (Fig. 4C), whereas NOD2 knockdown increased these abilities. In addition, Western blot analyzed the implications of NOD2 in the Epithelial-Mesenchymal Transition process. The results revealed that NOD2 overexpression decreased MMP2, MMP9, N-cadherin, and vimentin while increasing E-cadherin expression. The opposite result was obtained after NOD2 knockdown (Fig. 4D).

### NOD2 inhibits human melanoma growth in vivo

**Fig. 5:**
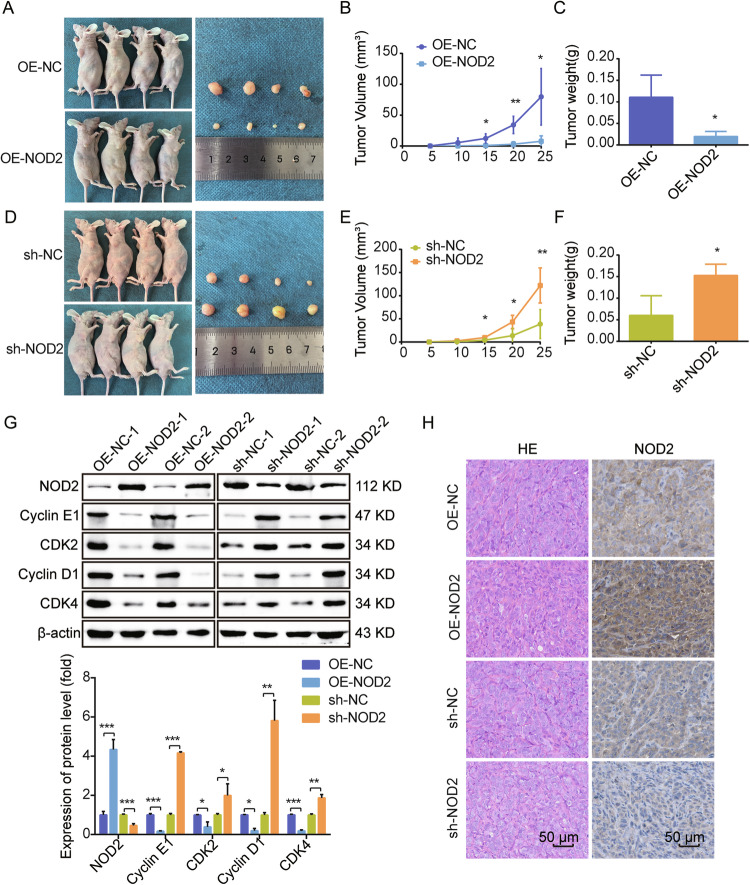
NOD2 inhibits human melanoma growth in vivo. **A**–**C** 1 × 10^7^ of A875 cells with NOD2 overexpression and control (OE-NC) were injected into the left (OE-NC) and right (OE-NOD2) sides of BALB/c nude mice (*n* = 4 per group). After tumor formation, mice were euthanized, and photographs of the tumors were taken (**A**). Tumor volume was monitored, and growth curves were plotted (**B**). Tumor weight was measured (**C**). **D**–**F** 1 × 10^7^ of A875 cells with NOD2 knockdown and control (sh-NC) were injected into the left (sh-NC) and right (sh-NOD2) sides of BALB/c nude mice (*n* = 4 per group). After tumor formation, mice were euthanized, and photographs of the tumors were taken (**D**). Tumor volume was monitored, and growth curves were plotted (**E**). Tumor weight was measured (**F**). **G** NOD2 expression and related cyclins were detected by Western blot. **H** HE staining and IHC staining of the tumor. Scale bar = 50 μm. Data are expressed as the mean ± SD. Student’s *t*-test and one-way ANOVA were used to compare the differences. *P < 0.05, **P < 0.01, ***P < 0.001.

To verify if targeting NOD2 inhibited melanoma development in vivo, we implanted A875 cells with NOD2 overexpression, knockdown, and its control group subcutaneously in nude mice to observe tumor formation. In contrast to the control group, the implanted NOD2 overexpression cell group more effectively suppressed the tumor growth rate and size (Fig. 5A–C). In contrast, accelerated proliferation and larger tumor growth were measured in the tumors implanted into the NOD2 knockdown group of cells (Fig. 5D–F). We also used another NOD2 knockdown sequence (sh-NOD2-1) of A875 cells injected subcutaneously into nude mice to reconfirm the promotional effect of NOD2 knockdown on melanoma cell growth in vivo (Supplementary Fig. 1A–C). Additionally, Western blot analysis was performed to determine NOD2 expression and its associated cyclin proteins in the tumor tissues of both groups. As depicted in Fig. 5G, the NOD2 overexpression group exhibited significantly higher levels of NOD2 expression in the tumor bodies compared to the control group. A downregulation of Cyclin E1, CDK2, Cyclin D1, and CDK4 accompanied this elevated NOD2 expression. The knockdown group displayed the opposite results. Also, the tumor was stained with Hematoxylin-eosin (HE) and immunohistochemical (IHC) (Fig. 5H).

### Inhibition of TYMS and PLK1 expression and activity is critical for the role of NOD2 in melanoma

**Fig. 6:**
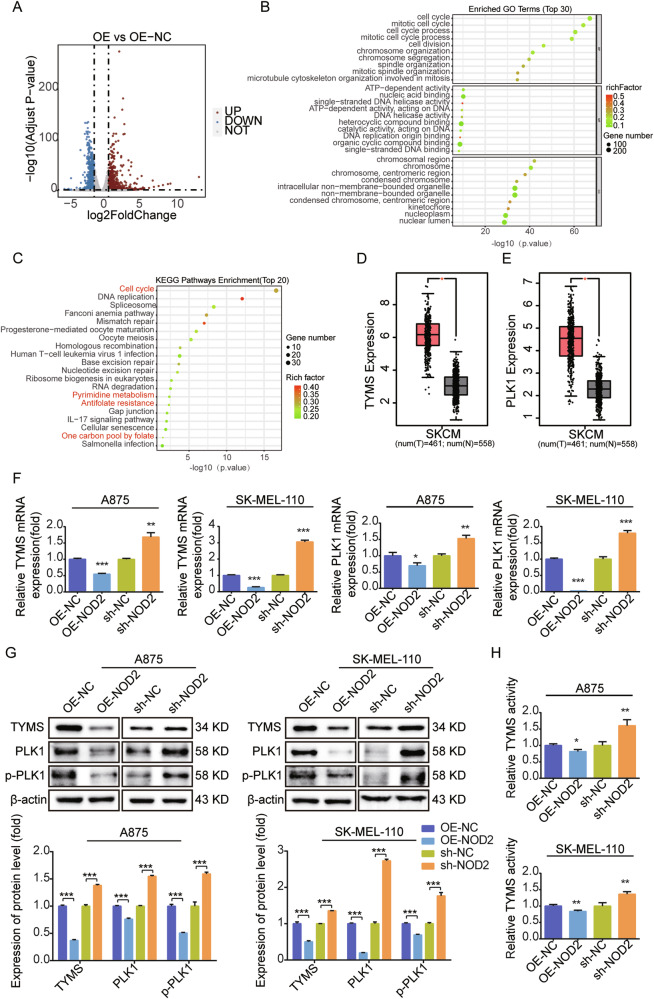
NOD2 negatively regulates the expression and activities of TYMS and PLK1. **A** Volcano plot of differential gene distribution after NOD2 overexpression in SK-MEL-110 melanoma cells. **B**, **C** GO analysis of the top 30 downregulated genes (**B**) and KEGG analysis of the top 20 downregulated genes (**C**) in NOD2 overexpressing SK-MEL-110 melanoma cells. **D**, **E** GEPIA database analysis of mRNA expression of TYMS and PLK1 in melanoma and normal tissues. **F**, **G** Real-time PCR determination of mRNA expression of TYMS, PLK1 after NOD2 overexpression and knockdown in A875 and SK-MEL-110 cells (**F**) and Western blot determination of protein levels of TYMS, PLK1, and p-PLK1 (**G**). **H** ELISA kit evaluates TYMS activity after NOD2 overexpression and knockdown in A875 and SK-MEL-110 cells. Data are expressed as the mean ± SD. Student’s *t*-test and one-way ANOVA were used to compare the differences. *P < 0.05, **P < 0.01, ***P < 0.001.

To investigate the biological function and mechanism of action of NOD2 in melanoma, we performed transcriptome sequencing of NOD2 overexpressing SK-MEL-110 melanoma cells. We identified 1583 differentially expressed genes, including 914 upregulated and 669 downregulated (Fig. 6A). We performed GO analysis on the top 30 downregulated genes (Fig. 6B) and KEGG enrichment analysis on the top 20 genes (Fig. 6C), with particular attention to the processes of the cell cycle, pyrimidine metabolism, antifolate resistance, and one-carbon pool by folate. PLK1 is a critical gene in the cell cycle [33], whereas TYMS plays essential roles in pyrimidine metabolism [34], antifolate resistance, and one-carbon pool by folate.

Using the GEPIA database, we found that the expression of TYMS and PLK1 were significantly upregulated in melanoma (Fig. 6D, E). Real-time PCR and Western blot results showed that NOD2 overexpression suppressed the mRNA and protein expression of TYMS and PLK1 (Fig. 6F, G). In addition, NOD2 overexpression decreased the expression of p-PLK1, the activated form of PLK1, whereas NOD2 knockdown increased p-PLK1 expression (Fig. 6G). Melanoma cells and an in vivo model of sh-NOD2-1, we reconfirmed that NOD2 knockdown upregulated the protein levels of TYMS, PLK1, and p-PLK1 (Supplementary Fig. 1D, E). Detection of the enzymatic activity of TYMS revealed that NOD2 overexpression inhibited its activity, whereas NOD2 knockdown enhanced its activity (Fig. 6H). NOD2 may function in melanoma by regulating the expression and activities of TYMS and PLK1.

### NOD2 negatively regulates TYMS protein abundance by promoting the K48-linked ubiquitination of TYMS

**Fig. 7:**
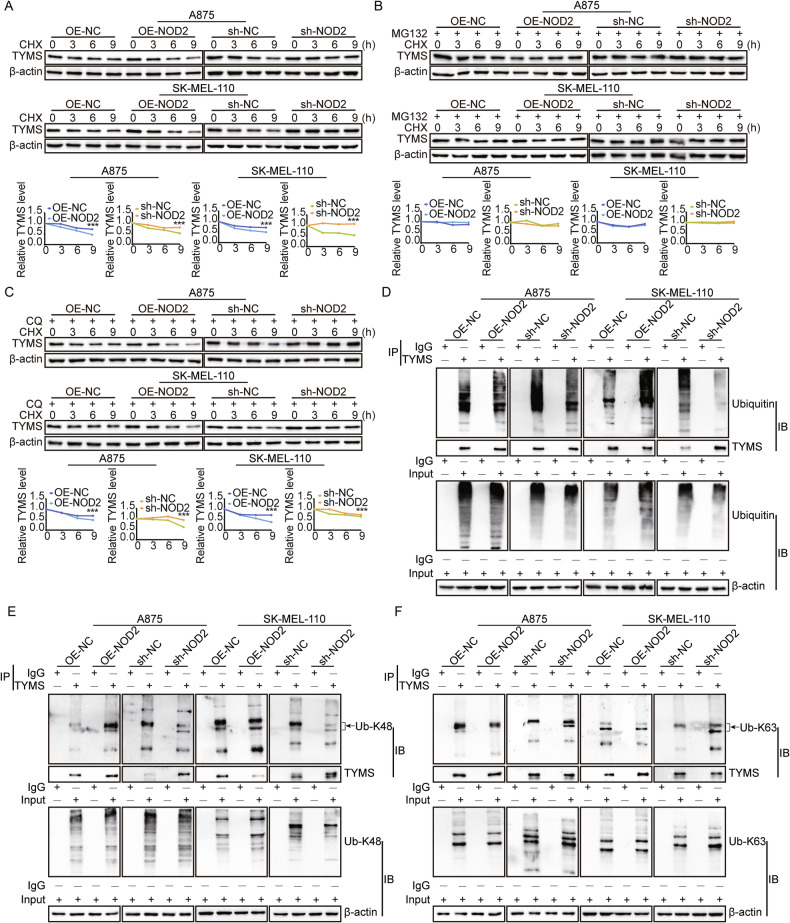
NOD2 negatively regulates TYMS protein abundance by promoting the K48-linked ubiquitination of TYMS. **A**–**C** Western blot detection of protein levels of TYMS in stably transfected A875 and SK-MEL-110 cells treated with cycloheximide (CHX) (100 μg/mL) with or without MG-132 (10 μg/mL) and CQ (20 μM) for 0, 3, 6, and 9 h. **D** CO-IP assay detected the ubiquitination expression level of TYMS in stably transfected A875 and SK-MEL-110 cells. **E**, **F** CO-IP assay detected the K48-linked (**E**) and K63-linked (**F**) ubiquitination expression levels of TYMS in stably transfected A875 and SK-MEL-110 cells. Data are expressed as the mean ± SD. Student’s *t*-test and one-way ANOVA were used to compare the differences, ***P < 0.001.

Our results (Fig. 6) showed that NOD2 regulates TYMS expression at the mRNA level and significantly affects its protein level. To explore the regulatory mechanism of NOD2 on TYMS degradation, we treated A875 and SK-MEL-110 cells with cycloheximide (CHX) under NOD2 knockdown and overexpression. We introduced the proteasome inhibitor MG-132 and the autophagy inhibitor chloroquine (CQ). The results showed that NOD2 overexpression accelerated TYMS degradation, whereas NOD2 knockdown significantly slowed the degradation (Fig. 7A). CHX reversed the degradation effect in combination with MG-132 treatment (Fig. 7B), but this change was not seen with CQ treatment (Fig. 7C), suggesting that NOD2 facilitates TYMS degradation through the proteasome pathway.

In addition, NOD2 overexpression increased the ubiquitination level of TYMS, while NOD2 knockdown decreased it (Fig. 7D). Lysine 48 (K48)-linked polyubiquitination usually leads to proteasome-mediated degradation, whereas lysine 63 (K63)-linked polyubiquitination has been associated with cell signaling and DNA repair. Our study found that NOD2 predominantly increased K48-linked ubiquitin chains on TYMS (Fig. 7E) while decreasing K63-linked ubiquitin chains (Fig. 7F). These findings suggest that the ubiquitination of TYMS is a complex and inconsistent process. Previous studies have also indicated that K48 and K63 ubiquitin chains can coexist and competitively attach to downstream proteins to regulate their stability [35]. In conclusion, NOD2 negatively regulates the protein abundance of TYMS mainly by increasing K48-linked ubiquitin chains on TYMS.

### TYMS regulates PLK1 expression and activation through interaction with PLK1

**Fig. 8:**
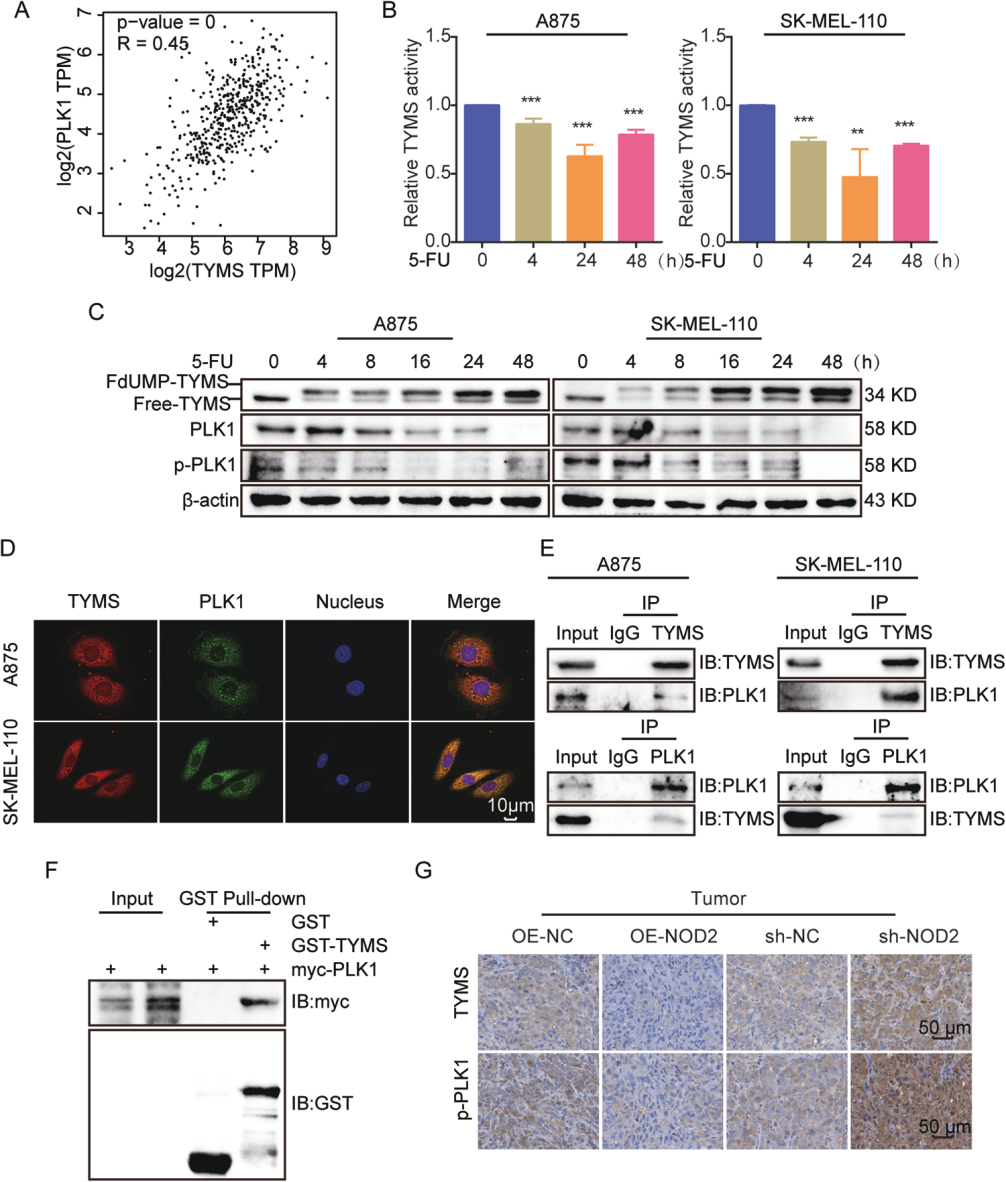
TYMS regulates PLK1 expression and activation through interaction with PLK1. **A** Correlation analysis of TYMS and PLK1 in melanoma in the GEPIA database. **B** A875 and SK-MEL-110 cells were treated with 5-FU (5 μg/ml) for 0, 4, 24, and 48 h, then evaluated for TYMS activity by ELISA kit. **C** A875 and SK-MEL-110 cells were treated with 5-FU (5 μg/ml) for 0, 4, 8, 16, 24, and 48 h. Western blot assays to detect the expression of TYMS, PLK1, and p-PLK1. **D** IF assay to analyze the localization of TYMS and PLK1 in A875 and SK-MEL-110 cells. Scale = 10 μm. **E** CO-IP assay to detect the interaction between TYMS and PLK1 in A875 and SK-MEL-110 cells. **F** GST Pull-down assay detects direct interaction between TYMS and PLK1. **G** IHC staining of TYMS and p-PLK1 in NOD2 overexpression and knockdown tumors. Scale = 50 μm. Data are expressed as the mean ± SD. Student’s *t*-test and one-way ANOVA were used to compare the differences. **P < 0.01, ***P < 0.001.

NOD2 may affect the biological behavior of melanoma cells by regulating the expression and activation status of TYMS and PLK1. Since PLK1 is a direct cell cycle checkpoint, we hypothesized that the regulation of melanoma cell behavior by NOD2 depends on changes in PLK1. Through GEPIA database analysis, we found a positive correlation between TYMS and PLK1 (Fig. 8A) and subsequently validated it. After using the TYMS inhibitor 5-FU [24], we observed a decrease in TYMS activity at 4 and 24 h. However, it was elevated again after 48 h (Fig. 8B), possibly due to an increased cellular resistance to 5-FU. 5-FU treatment resulted in two forms of TYMS [36]: most of it was captured in an inactive form by its metabolite 5-fluorodeoxyuridine monophosphate (FdUMP) (FdUMP-TYMS) and a small amount of free-TYMS in an active form (Free-TYMS). With the prolongation of 5-FU treatment, FdUMP-TYMS gradually increased, while Free-TYMS significantly decreased before 48 h and then increased after 48 h. The expression of PLK1 and p-PLK1 was also downregulated after 5-FU treatment (Fig. 8C), suggesting that PLK1 is a downstream effector molecule of TYMS, and TYMS positively regulates the expression and activity of PLK1.

Cellular immunofluorescence (IF) staining and co-immunoprecipitation (CO-IP) assays displayed that TYMS and PLK1 co-localized in the cytoplasm (Fig. 8D) and interacted with each other (Fig. 8E). GST Pull-down assays verified their direct interactions (Fig. 8F). IHC assays confirmed that in NOD2 overexpression or knockdown tumors, TYMS and p-PLK1 expression were negatively regulated by NOD2 (Fig. 8G). These results suggest that TYMS and PLK1 form a signaling axis in a direct reciprocal manner, and NOD2 exerts biological functions by regulating this signaling axis.

### NOD2 reduces chemoresistance targeting TYMS in melanoma

**Fig. 9:**
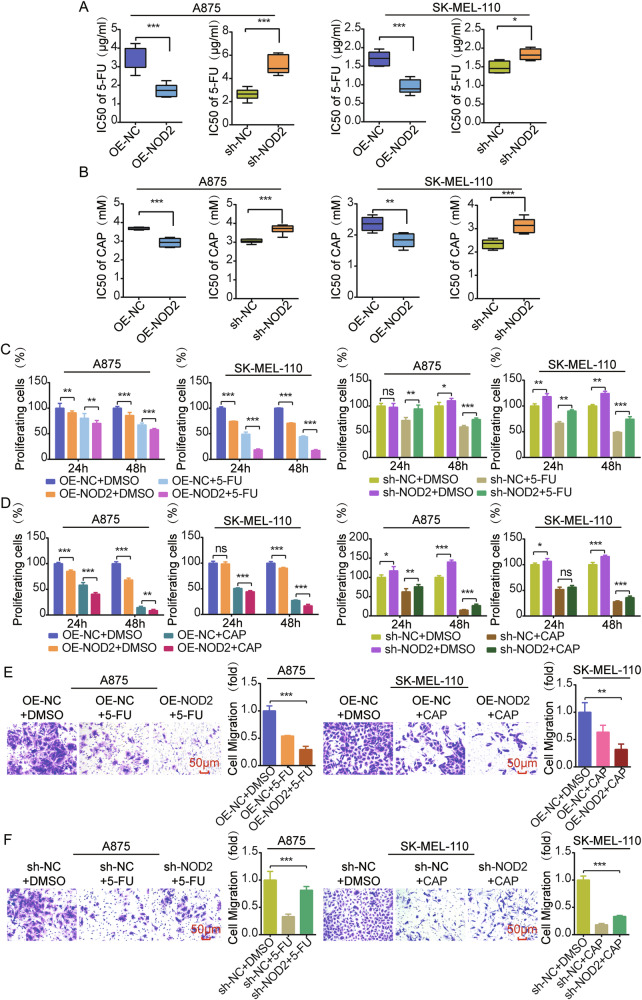
NOD2 reduces chemoresistance targeting TYMS in melanoma. **A**, **B** MTS assay to determine the IC50 values of NOD2 knockdown and overexpression in A875 and SK-MEL-110 cells after 48 h treatment with 5-FU (5 μg/ml) and CAP (2 mM). **C**, **D** MTS assay was performed to determine the proliferation of A875 and SK-MEL-110 cells with overexpression and knockdown of NOD2 after treatment with 5-FU and CAP. **E** Transwell migration assay was used to assess the migration ability of A875 cells with overexpression of NOD2 after 5-FU treatment and SK-MEL-110 cells with overexpression of NOD2 after CAP treatment. Scale = 50 μm. **F** Transwell migration assay was used to assess the migration ability of A875 cells with knockdown of NOD2 after 5-FU treatment and SK-MEL-110 cells with knockdown of NOD2 after CAP treatment. Scale = 50 μm. Data are expressed as the mean ± SD. Student’s *t*-test and one-way ANOVA were used to compare the differences. *P < 0.05, **P < 0.01, ***P < 0.001. *ns* not significant.

TYMS is an essential target of 5-FU [24] and some folate analogs [37], and its expression correlates with tumor chemoresistance. We hypothesized that NOD2 may influence chemoresistance in melanoma through the TYMS/PLK1 signaling axis. In NOD2 overexpressed A875 and SK-MEL-110 cells, the IC50 values of 5-FU and CAP were reduced, whereas in NOD2 knockdown cells, the IC50 values were elevated (Fig. 9A, B). NOD2 overexpressed cells exhibited more potent inhibition of cell proliferation in response to 5-FU or CAP treatment, whereas NOD2 knockdown cells were attenuated (Fig. 9C, D). In addition, NOD2 overexpression enhanced the inhibitory effect of 5-FU or CAP on cell migration (Fig. 9E), while NOD2 knockdown reversed this effect (Fig. 9F).

### Combination therapy targeting inhibition of TYMS and PLK1 suppresses melanoma progression

**Fig. 10:**
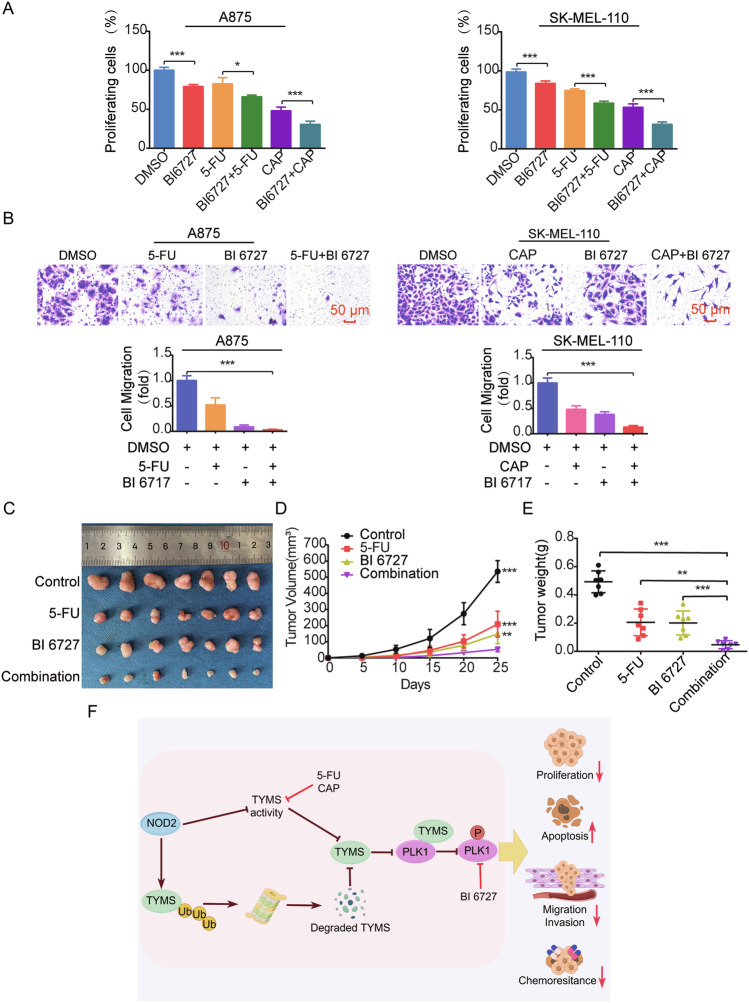
Combination therapy targeting inhibition of TYMS and PLK1 suppresses melanoma progression both in vitro and in vivo*.* **A** MTS assay was used to determine the proliferation of A875 and SK-MEL-110 cells after treatment with BI6727, 5-FU, and CAP, respectively, as well as combined treatment with BI6727 and 5-FU, BI6727, and CAP for 24 h. **B** Transwell migration assay was used to determine the 24-h cell migration capacity of A875 cells after treatment with BI6727 combined with 5-FU and SK-MEL-110 cells after treatment with BI6727 combined with CAP. 5-FU (5 μg/ml), CAP (2 mM), BI6727 (100 nM). Scale = 50 μm. **C**–**E** Nude mice were injected subcutaneously with 1 × 10^7^ of A875 cells. After tumor formation, they were injected intraperitoneally with saline, 5-FU (25 mg/kg), BI6727 (10 mg/kg), and combined 5-FU and BI6727, respectively (*n* = 7 per group). Mice were euthanized, and the tumors were peeled off and photographed (**C**). Tumor volumes were monitored, and growth curves were plotted (**D**). Tumor weights were measured (**E**). **F** Model diagram of the role and mechanism of NOD2 in melanoma. Data are expressed as the mean ± SD. Student’s *t*-test and one-way ANOVA were used to compare the differences. *P < 0.05, **P < 0.01, ***P < 0.001.

Melanoma is complex and prone to drug resistance, limiting the efficacy of monotherapy. We evaluated the effectiveness of using TYMS and PLK1 as therapeutic targets using the PLK1 inhibitor volasertib (BI6727), 5-FU, CAP alone, and BI6727 combined with 5-FU or CAP treated A875 and SK-MEL-110 cells. Results showed that BI6727 combined with 5-FU or CAP treatment exerted a synergistic inhibitory effect on cell proliferation (Fig. 10A) and. migration (Fig. 10B). In the nude mouse model, the tumor was significantly reduced in the group treated with 5-FU or BI6727, and the reduction was even more significant in the group treated with the combination of 5-FU and BI6727 (Fig. 10C–E).

Previous studies proposed that 5-FU [38] and CAP [39] affect autophagic flux, and PLK1 regulates autophagy [40]. Therefore, we examined the protein levels of the core autophagy proteins, Autophagy-related protein 7 (ATG7), Sequestosome 1 (SQSTM1/p62), BCL2 interacting protein 3 (BNIP3), and Microtubule-associated protein 1 light chain 3B (LC3B) to assess the changes in autophagic fluxes in those above cellular and animal models. The results showed that treatment with 5-FU, CAP, and BI6727 alone or in combination significantly downregulated ATG7 and upregulated SQSTM1/p62, BNIP3, and LC3II levels (Supplementary Fig. 2A). In addition, the autophagy inhibitor Chloroquine (CQ) effectively inhibited proliferation (Supplementary Fig. 2B) and migration (Supplementary Fig. 2C) of A875 and SK-MEL-110 cells. NOD2 overexpression combined with CQ reduced the proliferation (Supplementary Fig. 2D) and migration (Supplementary Fig. 2E) of cells to a greater extent, inhibiting melanoma cell progression.

In summary, combination therapy for melanoma cell progression is an effective therapeutic strategy that can enhance the therapeutic effect by targeting NOD2, TYMS, and PLK1.

## Discussion

The role of NOD2 in immune response [41] and inflammation [42, 43] has been extensively studied, but its role in tumors is unclear. Previous research has revealed that NOD2 functions as an immunosurveillance factor in certain types of cancers, such as colorectal [14] and esophageal adenocarcinomas [13], where it is considered protective. Conversely, aberrant activation of NOD2 promotes tumor progression in hepatocellular [16] and cervical [15] cancers. In this study, we investigated the role of NOD2 in melanoma, and the results showed that NOD2 has a protective function. The upregulation of NOD2 inhibited melanoma cell proliferation and migratory invasion while promoting apoptosis. Moreover, NOD2 upregulation reduced chemoresistance, enhancing its effectiveness in combating melanoma cells.

To understand the role of NOD2 in melanoma, we identified TYMS as a critical gene regulated by NOD2. TYMS is involved in DNA synthesis and cell proliferation processes [23]. Some chemotherapeutic agents targeting TYMS, such as 5-FU and CAP, treat tumors by inhibiting the biosynthetic activity of TYMS. TYMS expression and activity are associated with the malignant progression of tumors and resistance to chemotherapeutic drugs [29, 44–46]. Therefore, reducing TYMS expression and activity may inhibit melanoma malignant progression and reduce resistance to chemotherapeutic agents. TYMS expression is affected by multiple regulatory mechanisms. For example, the transcription factor FOXM1 can directly promote the transcription of TYMS, which can lead to the resistance of hepatocellular carcinoma cells to chemotherapeutic agents such as 5-FU [29]. Non-coding RNAs, such as miRNAs and lncRNAs, are also involved in the regulation of TYMS. MiR-330-5p [44] and miR-140-3p [45] inhibited tumor proliferation by suppressing TYMS expression. The long non-coding RNA SNHG15 promotes TYMS expression, leading to colorectal cancer resistance to 5-FU chemotherapeutic agents [46]. In addition, TYMS inhibits the growth of human hepatocellular carcinoma cells and triggers DNA damage through proteasome-dependent pathway degradation [47], but its ubiquitination site and type are mainly unknown.

In this study, we found that NOD2 negatively regulated TYMS expression and activity in melanoma and promoted its K48-linked ubiquitination, thereby enhancing the degradation of TYMS through the ubiquitin-proteasome system. Meanwhile, NOD2 inhibited the K63-linked ubiquitination of TYMS, probably due to the competition between the formation of K48 and K63 chains on TYMS during the dynamic assembly of ubiquitin chains [48]. However, the decrease in TYMS K63-linked ubiquitination was less significant than the change in its K48-linked ubiquitination, making the K48 type the main factor in stabilizing TYMS protein, while the K63 type also contributed to some extent. The study provided new insights into the regulatory mechanisms of TYMS, and further identification of specific proteasomal and ubiquitination sites that bind to TYMS is needed in the future. Our results indicate that overexpression of NOD2 could reduce the TYMS level and thus inhibit melanoma cell resistance to chemotherapeutic drugs. Moreover, activation of NOD2 combined with 5-FU or CAP synergistically inhibits melanoma proliferation and migration in vitro.

Understanding the regulatory mechanisms of TYMS and its interactions with other factors contributes to a deeper understanding of tumor biology. Our research showed that TYMS binding to PLK1 to form a complex directly regulates PLK1 expression and activity. The significance of this finding lies in the well-documented role of PLK1 in cell cycle regulation, mitosis [49], and tumorigenesis [50]. PLK1 is a driver protein in tumor DNA repair [51], cell death pathways [52], and epithelial-to-mesenchymal transition [53]. By disrupting cell cycle progression and inducing DNA damage, TYMS may alter PLK1 expression and activity, affecting processes such as proliferation, apoptosis, and EMT in melanoma. Although TYMS and PLK1 have been recognized as prognostic markers for certain tumors [54, 55], their specific roles and mechanisms of interaction remain unclear. Our study provides the first evidence that TYMS has a direct positive regulatory effect on PLK1 expression and activity.

PLK1 is an attractive target for overcoming chemoresistance and immune checkpoints in clinical cancer therapy [56, 57]. Studies have shown that PLK1 inhibitors, combined with radiotherapy and chemotherapeutic agents, exhibit excellent anticancer effects [58–61]. For instance, targeted inhibition of PLK1 has significantly hindered esophageal squamous cell carcinoma (ESCC) progression and reduced the resistance to doxorubicin, a chemotherapeutic drug [59]. Similarly, targeted inhibition of PLK1 inhibited the proliferation of laryngeal squamous cell carcinoma and decreased resistance to cisplatin [60]. To further explore the potential of the TYMS/PLK1 signaling axis in combination therapy, we have investigated the effects of combining a PLK1 inhibitor (BI6727) with 5-FU or CAP in melanoma. The results showed that the combination treatment significantly suppressed melanoma proliferation and migration in vitro and inhibited melanoma growth in vivo compared to the individual treatments of 5-FU, CAP, and BI6727. Given the intimate connection among autophagy, tumor progression, and chemotherapy resistance [62, 63], our research indicates that the combined treatment with 5-FU, CAP, and BI6727 suppresses autophagy in vitro and in vivo. Employing autophagy inhibitors or NOD2 overexpression in conjunction with autophagy inhibitors effectively curtailed melanoma progression, potentially serving as a mechanism by which TYMS and PLK1 influence melanoma. These findings highlight the effectiveness of inhibiting TYMS and PLK1 in impeding melanoma progression and reducing chemoresistance, suggesting that combination therapy has the potential to improve survival and overall outcomes for melanoma patients.

We elucidated the role of NOD2 in inhibiting melanoma progression and reducing chemoresistance. Our findings are consistent with previous studies, which reported that a combination of NOD2 agonists with interferon [64] and tumor microparticle vaccine [65] activated NOD2 signaling, generated an anti-tumor immune response, and inhibited melanoma growth. While most of the previous studies focused on the immunomodulatory role of NOD2, the present study analyzed its protective role in melanoma from a genetic perspective. NOD2 exhibits different mechanisms of action in various diseases. NOD2 inhibits inflammation by activating NF-κB and MAPK signaling pathways [14, 16]. In hepatocellular carcinoma, NOD2 activates AMPK, MAPK, NF-κB, STAT3, and ERK pathways and induces nuclear autophagy directly through Lamin A/C [16, 66, 67]. NOD2 acts by regulating the TYMS/PLK1 signaling axis in melanoma. NOD2 negatively regulates TYMS activity and maintains its protein stability by regulating K48 and K63 ubiquitination of TYMS. When TYMS expression and activity are altered, it affects PLK1 expression and activity by directly binding to PLK1. Furthermore, NOD2 overexpression in combination with TYMS inhibition and combination therapy targeting TYMS and PLK1 showed synergistic inhibition of melanoma proliferation and migration. These findings provide potential avenues for further research and the development of melanoma treatment strategies. However, the heterogeneity of NOD2 and its application in other tumors still requires further research and clinical validation to ensure the safety and efficacy of combination therapies.

## Supplementary information

Supplementary figure and table legends

Supplementary Table S1

Supplementary Table S2

Supplementary Figure 1

Supplementary Figure 2

Western blot

## Data Availability

We are committed to sharing research data upon reasonable request. All data and materials generated in this study are available from the corresponding author.

## References

[1] Siegel RL, Giaquinto AN, Jemal A. Cancer statistics, 2024. CA Cancer J Clin. 2024;74:12–49. 38230766 10.3322/caac.21820

[2] Mai R, Zhou S, Zhong W, Rong S, Cong Z, Li Y, et al. Therapeutic efficacy of combined BRAF and MEK inhibition in metastatic melanoma: a comprehensive network meta-analysis of randomized controlled trials. Oncotarget. 2015;6:28502–12. 26143635 10.18632/oncotarget.4375 PMC4695075

[3] Wang L, Leite de Oliveira R, Huijberts S, Bosdriesz E, Pencheva N, Brunen D, et al. An acquired vulnerability of drug-resistant melanoma with therapeutic potential. Cell. 2018;173:1413–25.e1414. 29754815 10.1016/j.cell.2018.04.012

[4] Cowey CL, Boyd M, Aguilar KM, Beeks A, Krepler C, Scherrer E. An observational study of drug utilization and associated outcomes among adult patients diagnosed with BRAF-mutant advanced melanoma treated with first-line anti-PD-1 monotherapies or BRAF/MEK inhibitors in a community-based oncology setting. Cancer Med. 2020;9:7863–78. 32871054 10.1002/cam4.3312 PMC7643646

[5] Ferrucci PF, Lens M, Cocorocchio E. Combined BRAF-targeted therapy with immunotherapy in BRAF-mutated advanced melanoma patients. Curr Oncol Rep. 2021;23:138. 34735635 10.1007/s11912-021-01134-7

[6] Nassar KW, Hintzsche JD, Bagby SM, Espinoza V, Langouët-Astrié C, Amato CM, et al. Targeting CDK4/6 represents a therapeutic vulnerability in acquired BRAF/MEK inhibitor-resistant melanoma. Mol Cancer Ther. 2021;20:2049–60. 34376578 10.1158/1535-7163.MCT-20-1126 PMC9768695

[7] Buchbinder EI, Giobbie-Hurder A, Ott PA. A phase I/II study of MCS110 with BRAF/MEK inhibition in patients with melanoma after progression on BRAF/MEK inhibition. Invest N Drugs. 2023;41:365–70. 10.1007/s10637-023-01364-5 37097370

[8] Inohara N, Chamaillard M, McDonald C, Nuñez G. NOD-LRR proteins: role in host-microbial interactions and inflammatory disease. Annu Rev Biochem. 2005;74:355–83. 15952891 10.1146/annurev.biochem.74.082803.133347

[9] Gao J, Zhao X, Hu S, Huang Z, Hu M, Jin S, et al. Gut microbial DL-endopeptidase alleviates Crohn’s disease via the NOD2 pathway. Cell Host Microbe. 2022;30:1435–49.e1439. 36049483 10.1016/j.chom.2022.08.002

[10] Luo X, Wang X, Huang S, Xu B, Luo S, Li Y, et al. Paeoniflorin ameliorates experimental colitis by inhibiting gram-positive bacteria-dependent MDP-NOD2 pathway. Int Immunopharmacol. 2021;90:107224. 33302036 10.1016/j.intimp.2020.107224

[11] Mao L, Dhar A, Meng G, Fuss I, Montgomery-Recht K, Yang Z, et al. Blau syndrome NOD2 mutations result in loss of NOD2 cross-regulatory function. Front Immunol. 2022;13:988862. 36189261 10.3389/fimmu.2022.988862 PMC9520668

[12] Wang G, Zhang C, Jiang F, Zhao M, Xie S, Liu X. NOD2-RIP2 signaling alleviates microglial ROS damage and pyroptosis via ULK1-mediated autophagy during Streptococcus pneumonia infection. Neurosci Lett. 2022;783:136743. 35716964 10.1016/j.neulet.2022.136743

[13] Li X, Liu S, Jin L, Ma Y, Liu T. NOD2 inhibits the proliferation of esophageal adenocarcinoma cells through autophagy. J Cancer Res Clin Oncol. 2023;149:639–52. 36316517 10.1007/s00432-022-04354-x PMC9931811

[14] Udden SMN, Peng L, Gan JL, Shelton JM, Malter JS, Hooper LV, et al. NOD2 suppresses colorectal tumorigenesis via downregulation of the TLR pathways. Cell Rep. 2017;19:2756–70. 28658623 10.1016/j.celrep.2017.05.084 PMC6032983

[15] Zhang Y, Li N, Yuan G, Yao H, Zhang D, Li N, et al. Upregulation of NOD1 and NOD2 contribute to cancer progression through the positive regulation of tumorigenicity and metastasis in human squamous cervical cancer. BMC Med. 2022;20:55. 35130902 10.1186/s12916-022-02248-w PMC8822783

[16] Zhou Y, Hu L, Tang W, Li D, Ma L, Liu H, et al. Hepatic NOD2 promotes hepatocarcinogenesis via a RIP2-mediated proinflammatory response and a novel nuclear autophagy-mediated DNA damage mechanism. J Hematol Oncol. 2021;14:9. 33413510 10.1186/s13045-020-01028-4 PMC7791875

[17] Ghareghani M, Rivest S. The synergistic potential of combining PD-1/PD-L1 immune checkpoint inhibitors with NOD2 agonists in Alzheimer’s disease treatment. Int J Mol Sci. 2023;24:10905. 10.3390/ijms241310905 PMC10341981 37446081

[18] Guzelj S, Weiss M, Slütter B, Frkanec R, Jakopin Ž. Covalently conjugated NOD2/TLR7 agonists are potent and versatile immune potentiators. J Med Chem. 2022;65:15085–101. 36335509 10.1021/acs.jmedchem.2c00808 PMC9706565

[19] Dong Y, Wang S, Wang C, Li Z, Ma Y, Liu G. Antagonizing NOD2 signaling with conjugates of paclitaxel and muramyl dipeptide derivatives sensitizes paclitaxel therapy and significantly prevents tumor metastasis. J Med Chem. 2017;60:1219–24. 28075581 10.1021/acs.jmedchem.6b01704

[20] Weekes CD, Nallapareddy S, Rudek MA, Norris-Kirby A, Laheru D, Jimeno A, et al. Thymidylate synthase (TYMS) enhancer region genotype-directed phase II trial of oral capecitabine for 2nd line treatment of advanced pancreatic cancer. Invest N Drugs. 2011;29:1057–65. 10.1007/s10637-010-9413-7 PMC3110647 20306339

[21] Zhao M, Tan B, Dai X, Shao Y, He Q, Yang B, et al. DHFR/TYMS are positive regulators of glioma cell growth and modulate chemo-sensitivity to temozolomide. Eur J Pharm. 2019;863:172665. 10.1016/j.ejphar.2019.172665 31542479

[22] Kumar A, Singh AK, Singh H, Thareja S, Kumar P. Regulation of thymidylate synthase: an approach to overcome 5-FU resistance in colorectal cancer. Med Oncol. 2022;40:3. 36308643 10.1007/s12032-022-01864-z

[23] Carreras CW, Santi DV. The catalytic mechanism and structure of thymidylate synthase. Annu Rev Biochem. 1995;64:721–62. 7574499 10.1146/annurev.bi.64.070195.003445

[24] Longley DB, Harkin DP, Johnston PG. 5-fluorouracil: mechanisms of action and clinical strategies. Nat Rev Cancer. 2003;3:330–8. 12724731 10.1038/nrc1074

[25] Tsukihara H, Tsunekuni K, Takechi T. Folic acid-metabolizing enzymes regulate the antitumor effect of 5-fluoro-2’-deoxyuridine in colorectal cancer cell lines. PLoS ONE. 2016;11:e0163961. 27685866 10.1371/journal.pone.0163961 PMC5042458

[26] Guijarro MV, Kellish PC, Dib PE, Paciaroni NG, Nawab A, Andring J, et al. First-in-class multifunctional TYMS nonclassical antifolate inhibitor with potent in vivo activity that prolongs survival. JCI Insight. 2023;8:158798. 10.1172/jci.insight.158798 PMC10386886 37097751

[27] Xu F, Ye ML, Zhang YP, Li WJ, Li MT, Wang HZ, et al. MicroRNA-375-3p enhances chemosensitivity to 5-fluorouracil by targeting thymidylate synthase in colorectal cancer. Cancer Sci. 2020;111:1528–41. 32073706 10.1111/cas.14356 PMC7226198

[28] Kim N, Yang C. Sodium butyrate inhibits the expression of thymidylate synthase and induces cell death in colorectal cancer cells. Int J Mol Sci. 2024;25:1572. 10.3390/ijms25031572 PMC10855029 38338851

[29] Wang L, Shi C, Yu J, Xu Y. FOXM1-induced TYMS upregulation promotes the progression of hepatocellular carcinoma. Cancer Cell Int. 2022;22:47. 35093082 10.1186/s12935-021-02372-2 PMC8801073

[30] Guijarro MV, Nawab A, Dib P, Burkett S, Luo X, Feely M, et al. TYMS promotes genomic instability and tumor progression in Ink4a/Arf null background. Oncogene. 2023;42:1926–39. 37106126 10.1038/s41388-023-02694-7 PMC10244171

[31] Gutteridge RE, Singh CK, Ndiaye MA, Ahmad N. Targeted knockdown of polo-like kinase 1 alters metabolic regulation in melanoma. Cancer Lett. 2017;394:13–21. 28235541 10.1016/j.canlet.2017.02.013 PMC5415376

[32] Su S, Chhabra G, Ndiaye MA, Singh CK, Ye T, Huang W, et al. PLK1 and NOTCH positively correlate in melanoma and their combined inhibition results in synergistic modulations of key melanoma pathways. Mol Cancer Ther. 2021;20:161–72. 33177155 10.1158/1535-7163.MCT-20-0654 PMC7790869

[33] Kalous J, Aleshkina D. Multiple roles of PLK1 in mitosis and meiosis. Cells. 2023;12:187. 10.3390/cells12010187 PMC9818836 36611980

[34] Anderson DD, Quintero CM, Stover PJ. Identification of a de novo thymidylate biosynthesis pathway in mammalian mitochondria. Proc Natl Acad Sci USA. 2011;108:15163–8. 21876188 10.1073/pnas.1103623108 PMC3174652

[35] Wang Z, Xia Y, Wang Y, Zhu R, Li H, Liu Y, et al. The E3 ligase TRIM26 suppresses ferroptosis through catalyzing K63-linked ubiquitination of GPX4 in glioma. Cell Death Dis. 2023;14:695. 37872147 10.1038/s41419-023-06222-z PMC10593845

[36] Kurasaka C, Nishizawa N, Ogino Y, Sato A. Trapping of 5-fluorodeoxyuridine monophosphate by thymidylate synthase confers resistance to 5-fluorouracil. ACS Omega. 2022;7:6046–52. 35224365 10.1021/acsomega.1c06394 PMC8868108

[37] Goldman ID, Zhao R. Molecular, biochemical, and cellular pharmacology of pemetrexed. Semin Oncol. 2002;29:3–17. 12571805 10.1053/sonc.2002.37461

[38] Zitkute V, Kukcinaviciute E, Jonusiene V, Starkuviene V, Sasnauskiene A. Differential effects of 5-fluorouracil and oxaliplatin on autophagy in chemoresistant colorectal cancer cells. J Cell Biochem. 2022;123:1103–15. 35490372 10.1002/jcb.30267

[39] Wang H, Yang R, Wang Z, Cao L, Kong D, Sun Q, et al. Metronomic capecitabine with rapamycin exerts an immunosuppressive effect by inducing ferroptosis of CD4(+) T cells after liver transplantation in rat. Int Immunopharmacol. 2023;124:110810. 37625370 10.1016/j.intimp.2023.110810

[40] Du Y, Shang Y, Qian Y, Guo Y, Chen S, Lin X, et al. Plk1 promotes renal tubulointerstitial fibrosis by targeting autophagy/lysosome axis. Cell Death Dis. 2023;14:571. 37640723 10.1038/s41419-023-06093-4 PMC10462727

[41] Napier RJ, Lee EJ, Davey MP, Vance EE, Furtado JM, Snow PE, et al. T cell-intrinsic role for Nod2 in protection against Th17-mediated uveitis. Nat Commun. 2020;11:5406. 33106495 10.1038/s41467-020-18961-0 PMC7589501

[42] Rochereau N, Roblin X, Michaud E, Gayet R, Chanut B, Jospin F, et al. NOD2 deficiency increases retrograde transport of secretory IgA complexes in Crohn’s disease. Nat Commun. 2021;12:261. 33431850 10.1038/s41467-020-20348-0 PMC7801705

[43] Watson A, Forbes Satter L, Reiland Sauceda A, Kellermayer R, Karam LB. NOD2 polymorphisms may direct a Crohn disease phenotype in patients with very early-onset inflammatory bowel disease. J Pediatr Gastroenterol Nutr. 2023;77:748–52. 37229767 10.1097/MPG.0000000000003846

[44] Karimi L, Jaberi M, Asadi M, Zarredar H, Zafari V, Bornehdeli S, et al. Significance of microRNA-330-5p/TYMS expression axis in the pathogenesis of colorectal tumorigenesis. J Gastrointest Cancer. 2022;53:965–70. 34651293 10.1007/s12029-021-00695-x

[45] Wan S, Liu Z, Chen Y, Mai Z, Jiang M, Di Q, et al. MicroRNA-140-3p represses the proliferation, migration, invasion and angiogenesis of lung adenocarcinoma cells via targeting TYMS (thymidylate synthetase). Bioengineered. 2021;12:11959–77. 34818974 10.1080/21655979.2021.2009422 PMC8810165

[46] Li M, Sun S, Bian Z, Yao S, Liu M, You X, et al. SNHG15 promotes chemoresistance and glycolysis in colorectal cancer. Pathol Res Pr. 2023;246:154480. 10.1016/j.prp.2023.154480 37148838

[47] Yang A, Wu Q, Chen Q, Yang J, Li H, Tao Y, et al. Cinobufagin restrains the growth and triggers DNA damage of human hepatocellular carcinoma cells via proteasome-dependent degradation of thymidylate synthase. Chem Biol Interact. 2022;360:109938. 35427566 10.1016/j.cbi.2022.109938

[48] Wu H, Lu XX, Wang JR, Yang TY, Li XM, He XS, et al. TRAF6 inhibits colorectal cancer metastasis through regulating selective autophagic CTNNB1/β-catenin degradation and is targeted for GSK3B/GSK3β-mediated phosphorylation and degradation. Autophagy. 2019;15:1506–22. 30806153 10.1080/15548627.2019.1586250 PMC6693460

[49] Dalvi PS, Macheleidt IF, Lim SY, Meemboor S, Müller M, Eischeid-Scholz H, et al. LSD1 inhibition attenuates tumor growth by disrupting PLK1 mitotic pathway. Mol Cancer Res. 2019;17:1326–37. 30760542 10.1158/1541-7786.MCR-18-0971

[50] Gheghiani L, Wang L, Zhang Y, Moore XTR, Zhang J, Smith SC, et al. PLK1 induces chromosomal instability and overrides cell-cycle checkpoints to drive tumorigenesis. Cancer Res. 2021;81:1293–307. 33376114 10.1158/0008-5472.CAN-20-1377 PMC8026515

[51] De Blasio C, Zonfrilli A, Franchitto M, Mariano G, Cialfi S, Verma N, et al. PLK1 targets NOTCH1 during DNA damage and mitotic progression. J Biol Chem. 2019;294:17941–50. 31597699 10.1074/jbc.RA119.009881 PMC6879332

[52] Li X, Chen G, Liu B, Tao Z, Wu Y, Zhang K, et al. PLK1 inhibition promotes apoptosis and DNA damage in glioma stem cells by regulating the nuclear translocation of YBX1. Cell Death Discov. 2023;9:68. 36805592 10.1038/s41420-023-01302-7 PMC9938146

[53] Shin SB, Jang HR, Xu R, Won JY, Yim H. Active PLK1-driven metastasis is amplified by TGF-β signaling that forms a positive feedback loop in non-small cell lung cancer. Oncogene. 2020;39:767–85. 31548612 10.1038/s41388-019-1023-z PMC6976524

[54] Zhang YQ, Yuan Y, Zhang J, Lin CY, Guo JL, Liu HS, et al. Evaluation of the roles and regulatory mechanisms of PD-1 target molecules in NSCLC progression. Ann Transl Med. 2021;9:1168. 34430609 10.21037/atm-21-2963 PMC8350711

[55] Reza MS, Hossen MA, Harun-Or-Roshid M, Siddika MA, Kabir MH, Mollah MNH. Metadata analysis to explore hub of the hub-genes highlighting their functions, pathways and regulators for cervical cancer diagnosis and therapies. Discov Oncol. 2022;13:79. 35994213 10.1007/s12672-022-00546-6 PMC9395557

[56] Zhang Z, Cheng L, Li J, Qiao Q, Karki A, Allison DB, et al. Targeting Plk1 sensitizes pancreatic cancer to immune checkpoint therapy. Cancer Res. 2022;82:3532–48. 35950917 10.1158/0008-5472.CAN-22-0018 PMC9532376

[57] Jang HR, Shin SB, Kim CH, Won JY, Xu R, Kim DE, et al. PLK1/vimentin signaling facilitates immune escape by recruiting Smad2/3 to PD-L1 promoter in metastatic lung adenocarcinoma. Cell Death Differ. 2021;28:2745–64. 33963314 10.1038/s41418-021-00781-4 PMC8408167

[58] Wang D, Veo B, Pierce A, Fosmire S, Madhavan K, Balakrishnan I, et al. A novel PLK1 inhibitor onvansertib effectively sensitizes MYC-driven medulloblastoma to radiotherapy. Neuro Oncol. 2022;24:414–26. 34477871 10.1093/neuonc/noab207 PMC8917408

[59] Zeng TT, Deng TH, Liu Z, Zhan JR, Ma YZ, Yan YY, et al. HN1L/AP-2γ/PLK1 signaling drives tumor progression and chemotherapy resistance in esophageal squamous cell carcinoma. Cell Death Dis. 2022;13:1026. 36476988 10.1038/s41419-022-05478-1 PMC9729194

[60] Gao W, Zhang Y, Luo H, Niu M, Zheng X, Hu W, et al. Targeting SKA3 suppresses the proliferation and chemoresistance of laryngeal squamous cell carcinoma via impairing PLK1-AKT axis-mediated glycolysis. Cell Death Dis. 2020;11:919. 33106477 10.1038/s41419-020-03104-6 PMC7589524

[61] Zhang S, Yu J, Tan X, Cheng S, Liu H, Li Z, et al. A novel L-shaped ortho-quinone analog as PLK1 inhibitor blocks prostate cancer cells in G(2) phase. Biochem Pharm. 2024;219:115960. 38049008 10.1016/j.bcp.2023.115960

[62] Liang Y, Chen B, Xu F, Long L, Ye F, Wang Y, et al. LncRNA PRBC induces autophagy to promote breast cancer progression through modulating PABPC1-mediated mRNA stabilization. Oncogene. 2024;43:1019–32. 38366145 10.1038/s41388-024-02971-z

[63] Li W, Zhou C, Yu L, Hou Z, Liu H, Kong L, et al. Tumor-derived lactate promotes resistance to bevacizumab treatment by facilitating autophagy enhancer protein RUBCNL expression through histone H3 lysine 18 lactylation (H3K18la) in colorectal cancer. Autophagy. 2024;20:114–30. 37615625 10.1080/15548627.2023.2249762 PMC10761097

[64] Fujimura T, Yamasaki K, Hidaka T, Ito Y, Aiba S. A synthetic NOD2 agonist, muramyl dipeptide (MDP)-Lys (L18) and IFN-β synergistically induce dendritic cell maturation with augmented IL-12 production and suppress melanoma growth. J Dermatol Sci. 2011;62:107–15. 21411292 10.1016/j.jdermsci.2011.02.002

[65] Dong W, Zhang H, Yin X, Liu Y, Chen D, Liang X, et al. Oral delivery of tumor microparticle vaccines activates NOD2 signaling pathway in ileac epithelium rendering potent antitumor T cell immunity. Oncoimmunology. 2017;6:e1282589. 28405506 10.1080/2162402X.2017.1282589 PMC5384362

[66] Ma X, Qiu Y, Sun Y, Zhu L, Zhao Y, Li T, et al. NOD2 inhibits tumorigenesis and increases chemosensitivity of hepatocellular carcinoma by targeting AMPK pathway. Cell Death Dis. 2020;11:174. 32144252 10.1038/s41419-020-2368-5 PMC7060316

[67] Gurses SA, Banskar S, Stewart C, Trimoski B, Dziarski R, Gupta D. Nod2 protects mice from inflammation and obesity-dependent liver cancer. Sci Rep. 2020;10:20519. 33239685 10.1038/s41598-020-77463-7 PMC7688964

