# Supplementary material for: NOD2 reduces the chemoresistance of melanoma by inhibiting the TYMS/PLK1 signaling axis
Source: Cell Death Dis. 2024 Oct 1;15(10):720. doi: 10.1038/s41419-024-07104-8 (PMC11445241; doi:10.1038/s41419-024-07104-8)
Supplement: Supplementary file 1 — Supplementary figure and table legends [file 41419_2024_7104_MOESM1_ESM.doc]

**Supplementary figure and table legends**

**Supplementary Figure 1** **NOD2 knockdown promotes melanoma growth *in vivo* and upregulates the protein expression of TYMS, PLK1, and p-PLK1**

(A-C) 1×10^7^ of A875 cells with NOD2 knockdown (sh-NOD2-1) and control (sh-NC) were injected subcutaneously into BALB/c nude mice (n = 4 per group). After tumor formation, mice were euthanized, and photographs of the tumors were taken (A). Tumor volumes were monitored, and growth curves were plotted (B). Tumor weights were measured (C).

(D) TYMS, PLK1, and p-PLK1 expression were detected in NOD2 knockdown (sh-NOD2-1) and control (sh-NC) cells by Western blot.

(E) NOD2, TYMS, PLK1, and p-PLK1 expression were detected in NOD2 knockdown (sh-NOD2-1) and control (sh-NC) tumors by Western blot. N1, N2, and N3 represent tumor samples derived from (A).

Data are expressed as the mean ± SD. Student’s t-test and one-way ANOVA were used to compare the differences. **P* < 0.05, ***P* < 0.01, ****P* < 0.001.

**Supplementary Figure 2** **Combination therapy targeting inhibition of TYMS and PLK1 suppresses melanoma autophagy**

(A) Western blot determined expression levels of autophagy-related proteins, including ATG7, p62, BNIP3, and LC3Ⅰ/Ⅱ in A875 and SK-MEL-110 cells after treatment with 5-FU (5 μg/ml) or CAP (2 mM) combined with BI6727 (100 nM) and tumors of BALB/c nude mice treated with 5-FU (25 mg/kg) combined with BI6727 (10 mg/kg).

(B) MTS assay was performed to determine the proliferation of A875 and SK-MEL-110 cells treated with CQ (20 μM).

(C) Transwell migration assay was used to assess the migration ability of A875 and SK-MEL-110 cells treated with CQ (20 μM). Scale=50 μm

(D) MTS assay was performed to determine the proliferation of A875 and SK-MEL-110 cells with overexpression of NOD2 after treatment with CQ (20 μM).

(E) Transwell migration assay was used to assess the migration ability of A875 and SK-MEL-110 cells with overexpression of NOD2 after treatment with CQ (20 μM).

Data are expressed as the mean ± SD. Student’s t-test and one-way ANOVA were used to compare the differences. ****P* < 0.001, *ns* not significant.

**Supplementary Table S1 Specific primer sequences used in Real-time PCR**

**Supplementary Table S2 Primary antibodies and secondary antibodies used in Western blot analysis**
