# Supplementary material for: NOD2 reduces the chemoresistance of melanoma by inhibiting the TYMS/PLK1 signaling axis
Source: Cell Death Dis. 2024 Oct 1;15(10):720. doi: 10.1038/s41419-024-07104-8 (PMC11445241; doi:10.1038/s41419-024-07104-8)
Supplement: Supplementary file 2 — Supplementary Table S1 [file 41419_2024_7104_MOESM2_ESM.doc]

Supplementary Table S1 Specific primer sequences used in Real-time PCR

| Gene | primer |
| --- | --- |
| NOD2 | F: 5’- 3’: TGGTTCAGCCTCTCACGATGA  R: 5’- 3’: CAGGACACTCTCGAAGCCTT |
| TYMS | F: 5’- 3’: CTGCTGACAACCAAACGTGTG  R: 5’- 3’: GCATCCCAGATTTTCACTCCCTT |
| PLK1 | F: 5’- 3’: AAGTGGGTGGACTATTCG  R: 5’- 3’: GCCGTCACGCTCTATGTA |
| Cyclin E1 | F: 5’- 3’: ACTCAACGTGCAAGCCTCG  R: 5’- 3’: GCTCAAGAAAGTGCTGATCCC |
| CDK2 | F: 5’- 3’: CCAGGAGTTACTTCTATGCCTGA  R: 5’- 3’: TTCATCCAGGGGAGGTACAAC |
| Cyclin D1 | F: 5’- 3’: GCTGCGAAGTGGAAACCATC  R: 5’- 3’: CCTCCTTCTGCACACATTTGAA |
| CDK4 | F: 5’- 3’: TCAGCCAGCTTGACTGTTCCA  R: 5’- 3’: GCCTAGATTTCCTTCATGCCA |
| P16 | F: 5’- 3’: GGGTTTTCGTGGTTCACATCC  R: 5’- 3’: CTAGACGCTGGCTCCTCAGTA |
| Bax | F: 5’- 3’: CCCGAGAGGTCTTTTTCCGAG  R: 5’- 3’: CCAGCCCATGATGGTTCTGAT |
| Bcl2 | F: 5’- 3’: GGTGGGGTCATGTGTGTGG  R: 5’- 3’: CGGTTCAGGTACTCAGTCATCC |
| Caspase 3 | F: 5’- 3’: CATGGAAGCGAATCAATGGACT  R: 5’- 3’: CTGTACCAGACCGAGATGTCA |
| U6 | F: 5’- 3’:CTCGCTTCGGCAGCACA  R: 5’- 3’: AACGCTTCACGAATTTGCGT |
