# Supplementary material for: NOD2 reduces the chemoresistance of melanoma by inhibiting the TYMS/PLK1 signaling axis
Source: Cell Death Dis. 2024 Oct 1;15(10):720. doi: 10.1038/s41419-024-07104-8 (PMC11445241; doi:10.1038/s41419-024-07104-8)
Supplement: Supplementary file 3 — Supplementary Table S2 [file 41419_2024_7104_MOESM3_ESM.doc]

Supplementary Table S2 Primary antibodies and secondary antibodies used in Western blot analysis

| **Antibodies** | **Corporations** | **Catalog** | **Dilutions** |
| --- | --- | --- | --- |
| NOD2 | Abcam | #ab36836 | 1:2,000 |
| TYMS | Proteintech | #15047-1-AP | 1:2,000 |
| PLK1 | Santa Cruz | #sc-17783 | 1:500 |
| p-PLK1(Thr210) | Abcam | #ab155095 | 1:1,000 |
| Cyclin E1 | Proteintech | #11554-1-AP | 1:1,000 |
| Cyclin D1 | Proteintech | #60186-1-Ig | 1:1,000 |
| CDK2 | Proteintech | #10122-1-AP | 1:1,000 |
| CDK4 | Proteintech | #11026-1-AP | 1:1,000 |
| P27 | Proteintech | #25614-1-AP | 1:1,000 |
| Bcl2 | Proteintech | #68103-1-Ig | 1:1,000 |
| p-Bcl2(Ser70) | Proteintech | #80771-2-RR | 1:1,000 |
| Bax | Proteintech | #60267-1-Ig | 1:1,000 |
| Caspase 3 | Proteintech | #66470-2-Ig | 1:1,000 |
| MMP2 | Proteintech | #10373-2-AP | 1:1,000 |
| MMP9 | Proteintech | #10375-2-AP | 1:1,000 |
| N-cadherin | Proteintech | #22018-1-AP | 1:1,000 |
| E-Cadherin | Proteintech | #20874-1-AP | 1:1,000 |
| vimentin | Proteintech | #60330-1-Ig | 1:1,000 |
| Ubiquitin | CST | #43124 | 1:1,000 |
| Ubiquitin (linkage-specific K48) | Abcam | #ab140601 | 1:1,000 |
| Ubiquitin (linkage-specific K63) | Abcam | #ab179434 | 1:1,000 |
| ATG7 | Proteintech | #10088-2-AP | 1:1,000 |
| SQSTM1/p62 | HUABIO | #HA721171 | 1:2,000 |
| BNIP3 | Zenbio | #381756 | 1:500 |
| LC3B | HUABIO | #ET1701-65 | 1:1,000 |
| Myc-tag | MCE | HY-P80232 | 1:1,000 |
| GST-Tag | MCE | HY-P80148 | 1:1,000 |
| β-actin | Proteintech | #66009-1-Ig | 1:5,000 |
| rabbit IgG | CST | #7074 | 1:10,000 |
| mouse IgG | Santa Cruz | #sc2005 | 1:10,000 |
