# Supplementary figures and images for: NOD2 reduces the chemoresistance of melanoma by inhibiting the TYMS/PLK1 signaling axis
Source: Cell Death Dis. 2024 Oct 1;15(10):720. doi: 10.1038/s41419-024-07104-8 (PMC11445241; doi:10.1038/s41419-024-07104-8)

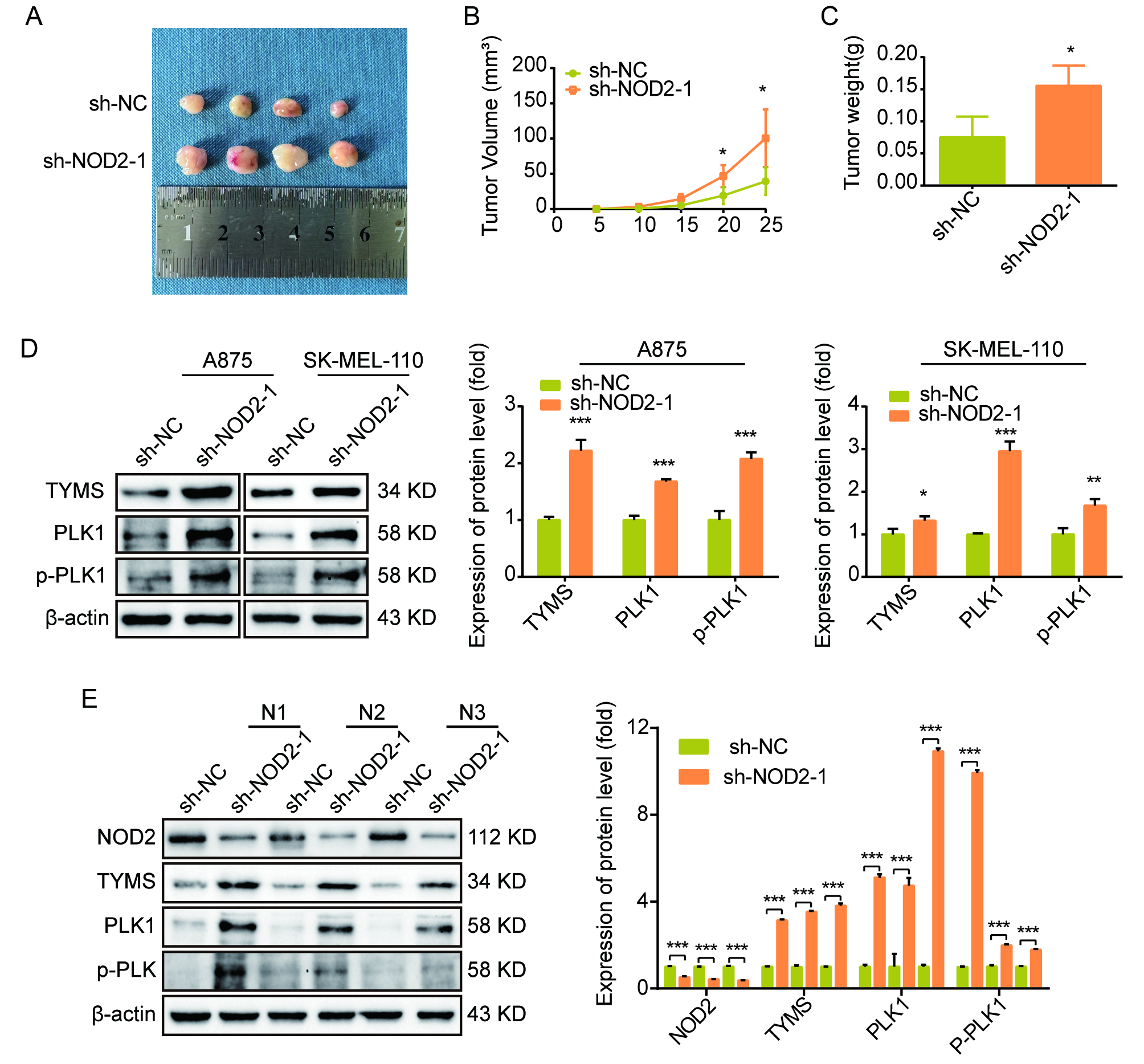

Supplement: Supplementary file 4 — Supplementary Figure 1 [file 41419_2024_7104_MOESM4_ESM.tif]
