# Supplementary material for: NOD2 reduces the chemoresistance of melanoma by inhibiting the TYMS/PLK1 signaling axis
Source: Cell Death Dis. 2024 Oct 1;15(10):720. doi: 10.1038/s41419-024-07104-8 (PMC11445241; doi:10.1038/s41419-024-07104-8)

Figure 1E

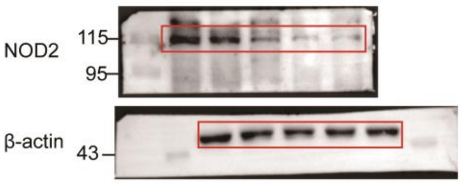

Figure 1F

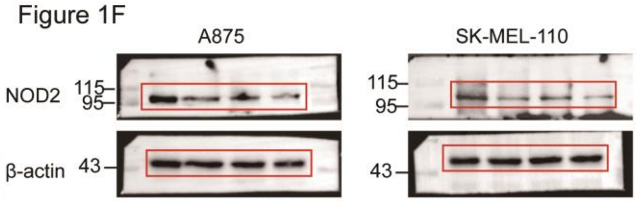

Figure 1G

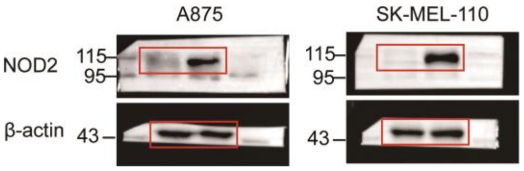

Figure 2F

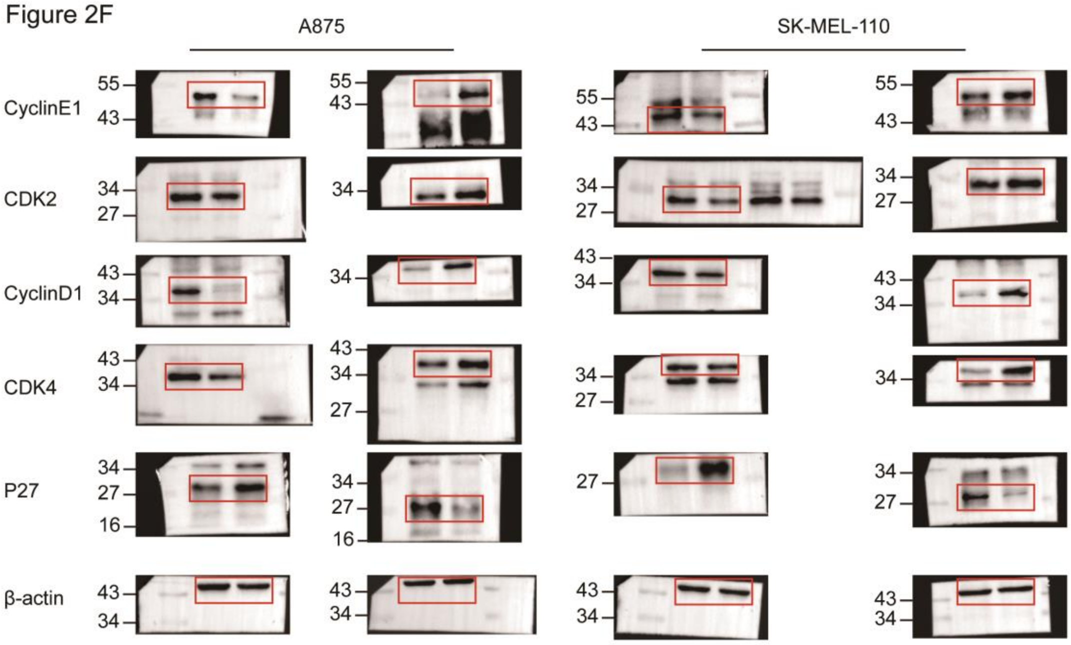

Figure 3C

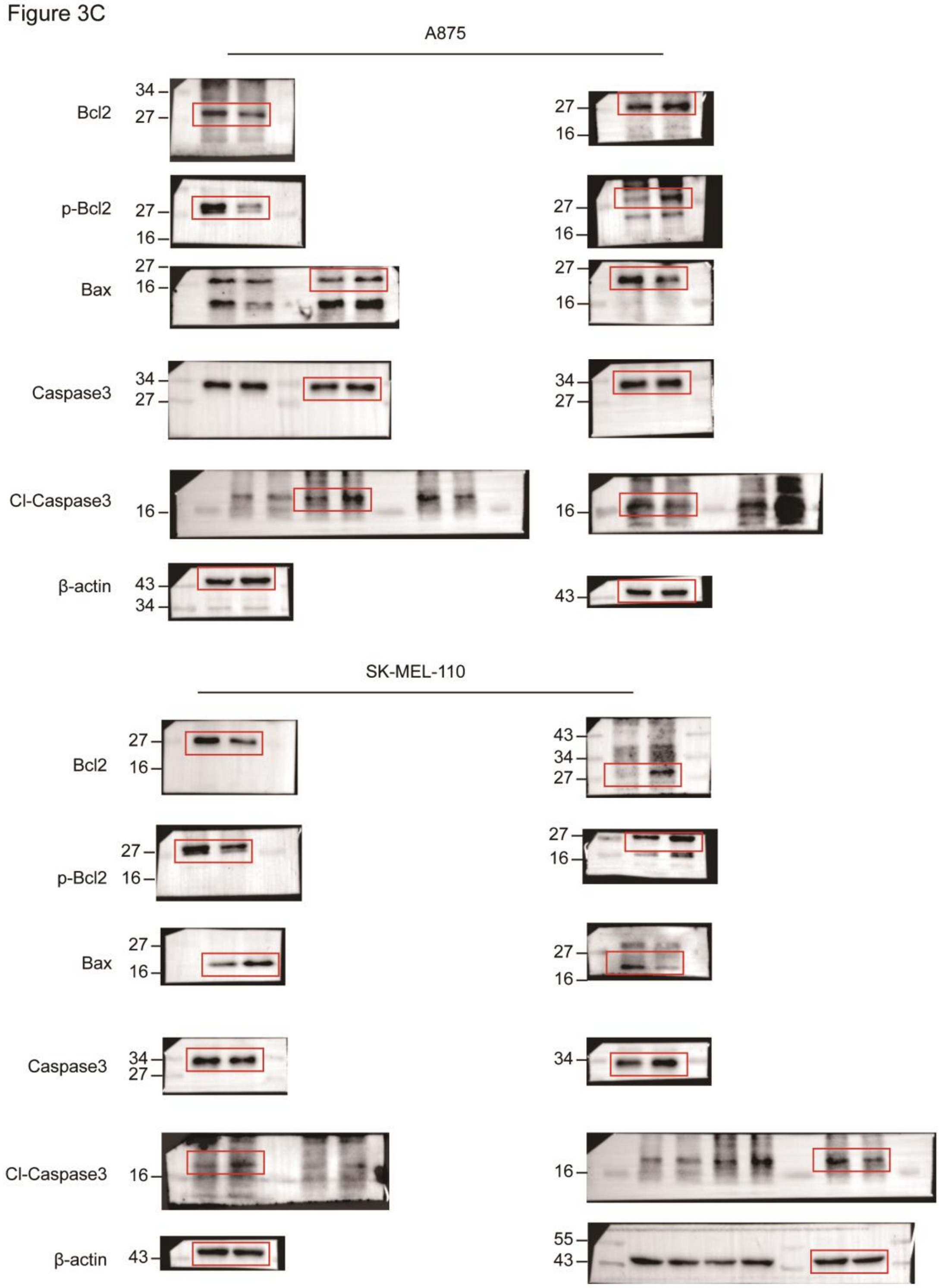
A875
Bcl2
34
27
27
16
p-Bcl2
27
16
27
16
Bax
27
16
27
16
Caspase3
34
27
34
27
Cl-Caspase3
16
16
β-actin
43
34
43
SK-MEL-110
Bcl2
27
16
43
34
27
p-Bcl2
27
16
27
16
Bax
27
16
27
16
Caspase3
34
27
34
Cl-Caspase3
16
16
β-actin
43
55
43

Figure 4D

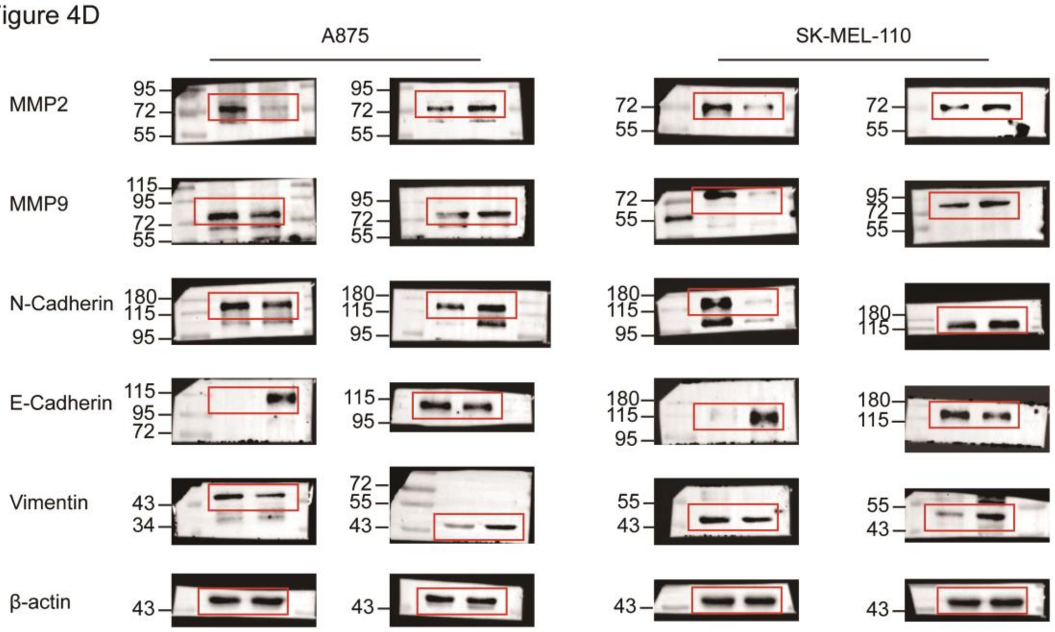

Figure 5G

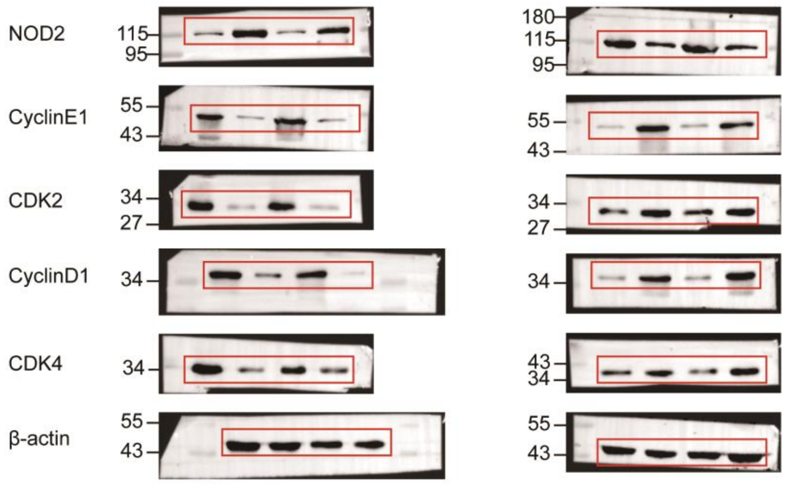

Figure 6G

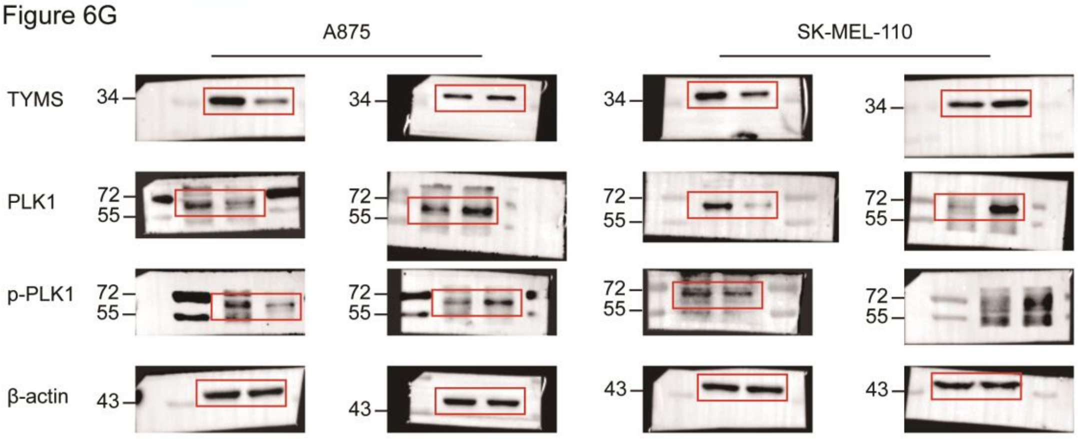

Figure 7A

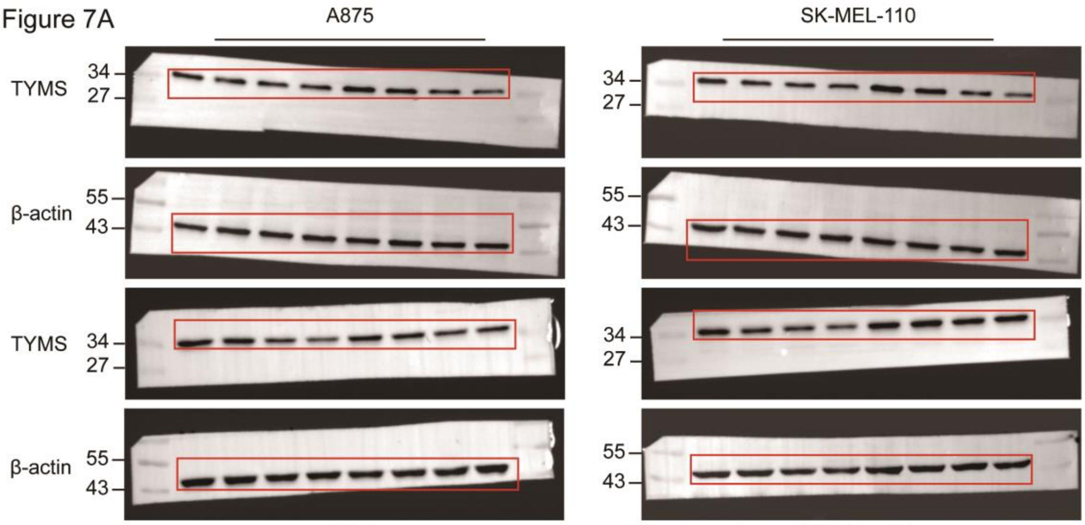

Figure 7B

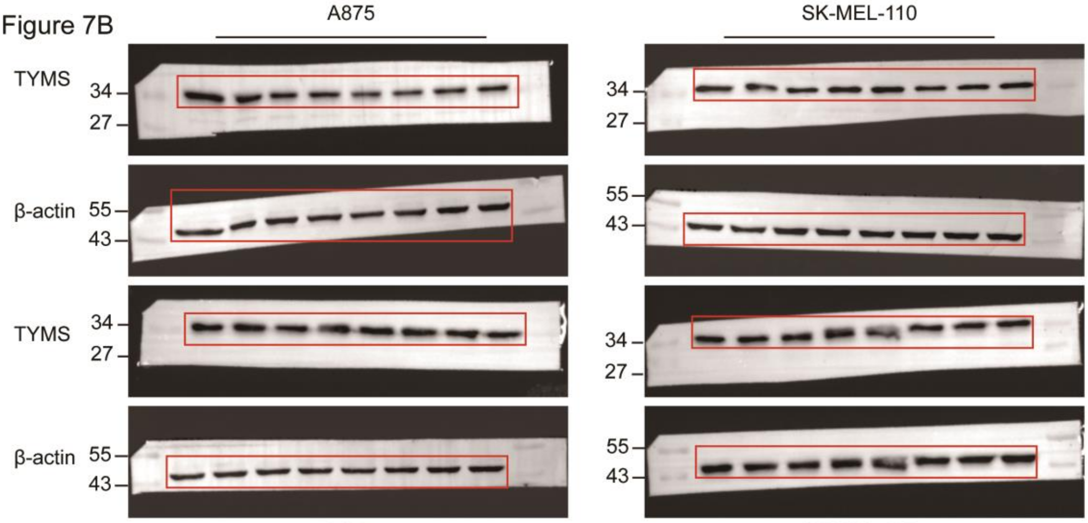

Figure 7C

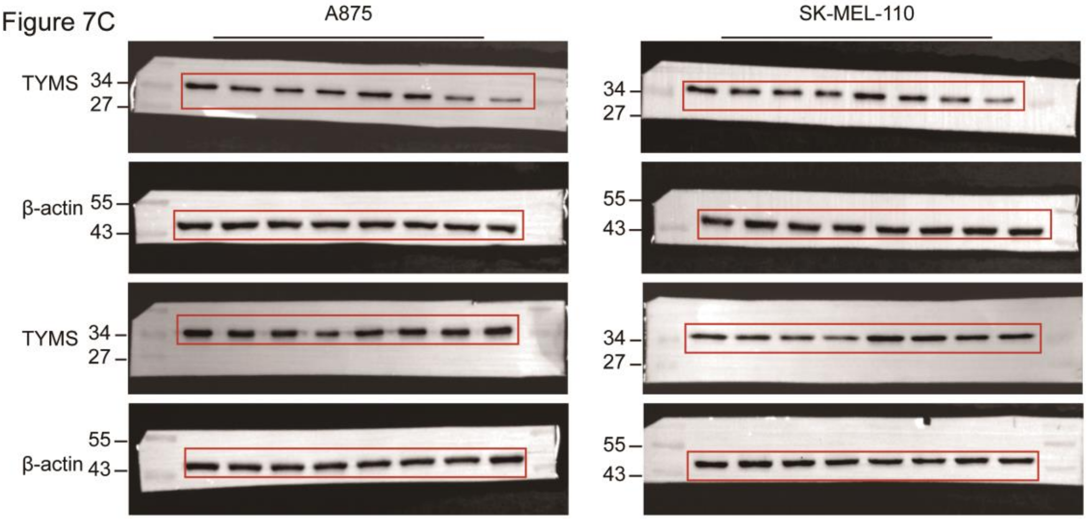

Figure 7D

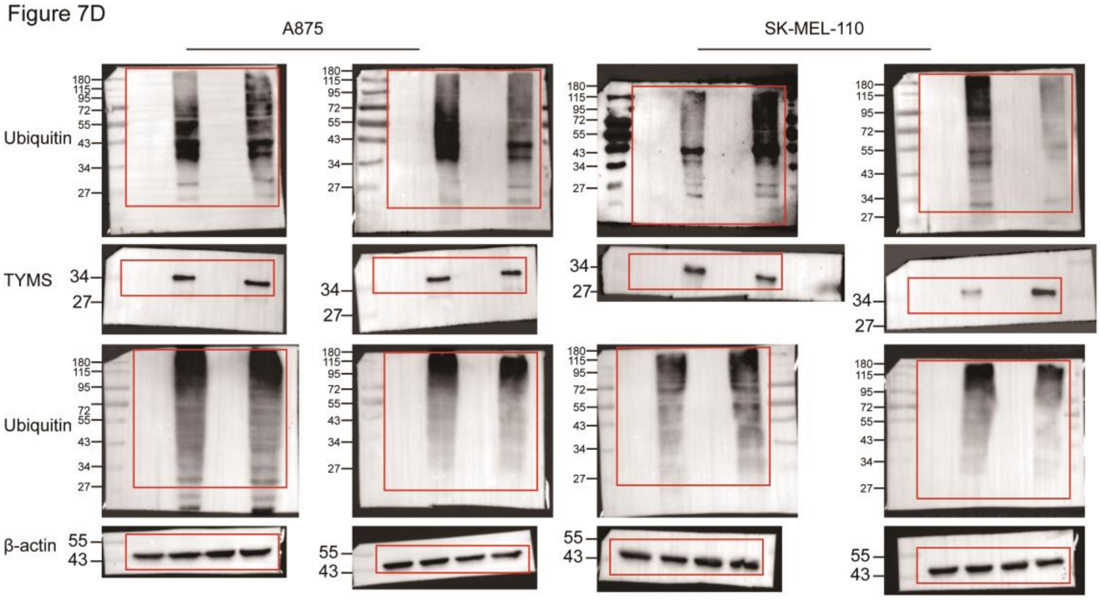

Figure 7E

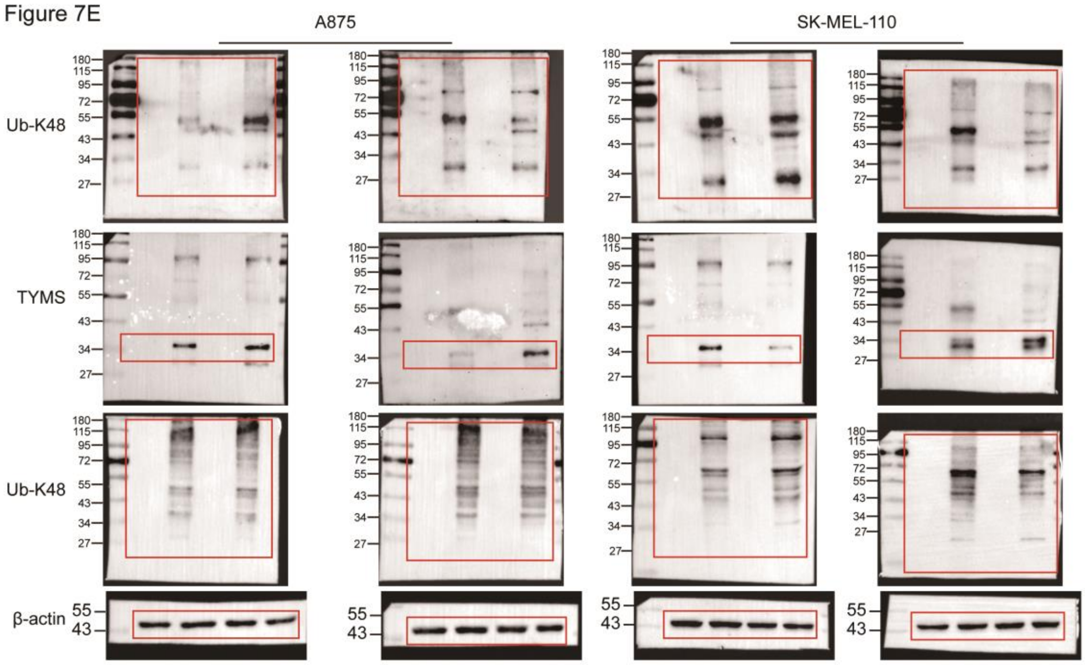

Figure 7F

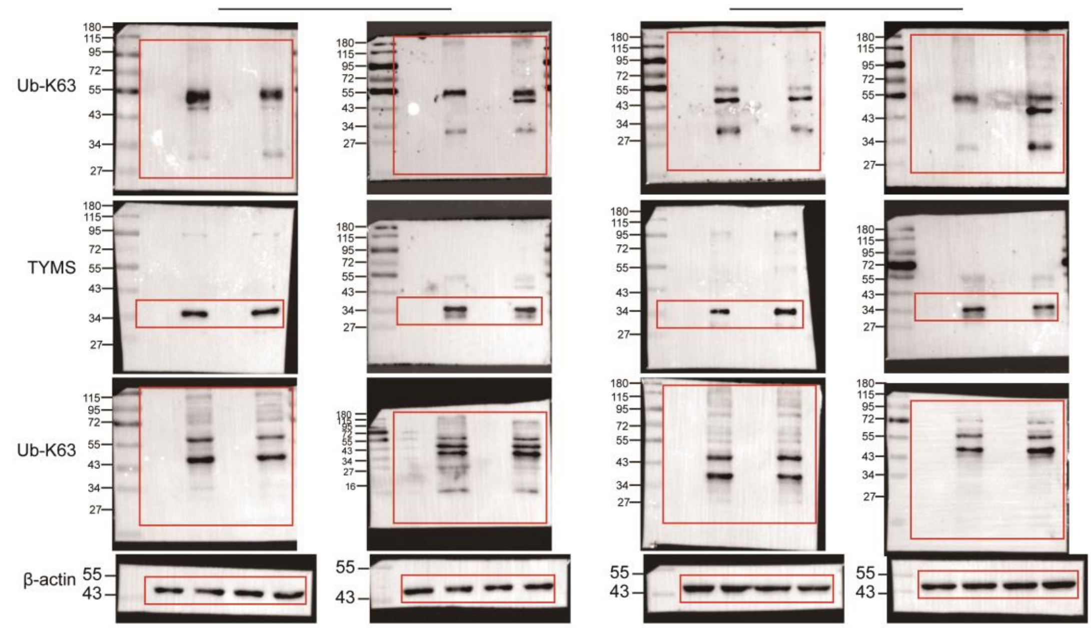

Figure 8C

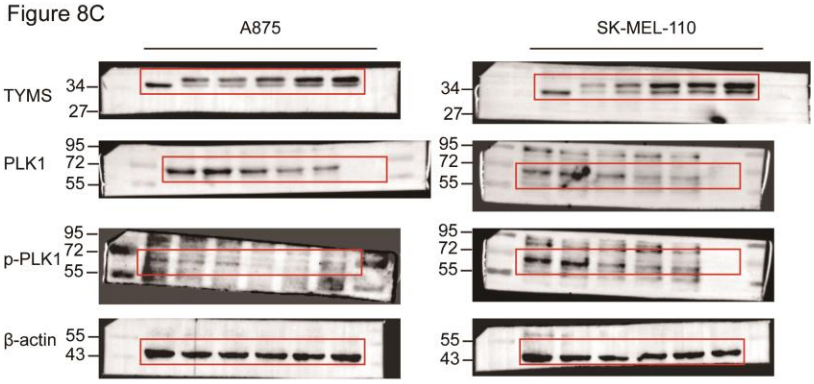

Figure 8F

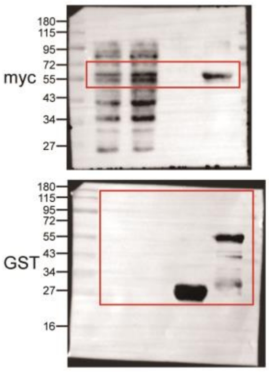

Figure 8E

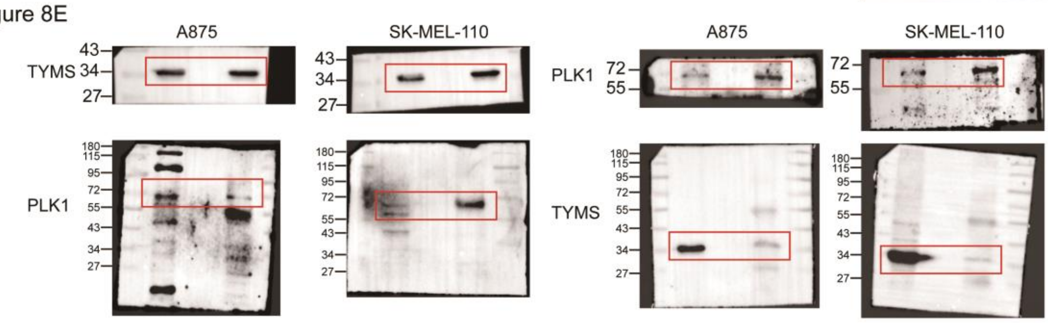

Supplementary Figure 1D

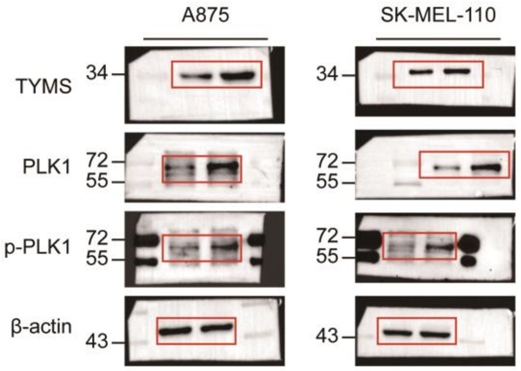

Supplementary Figure 1E

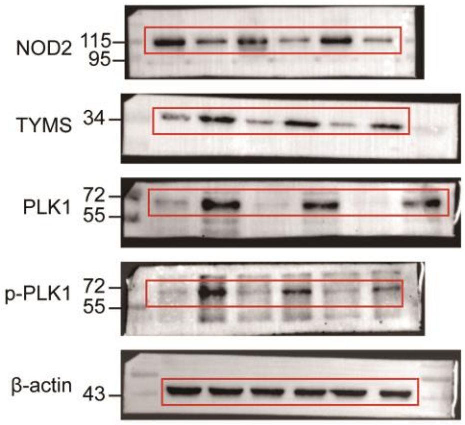

Supplementary Figure 2A

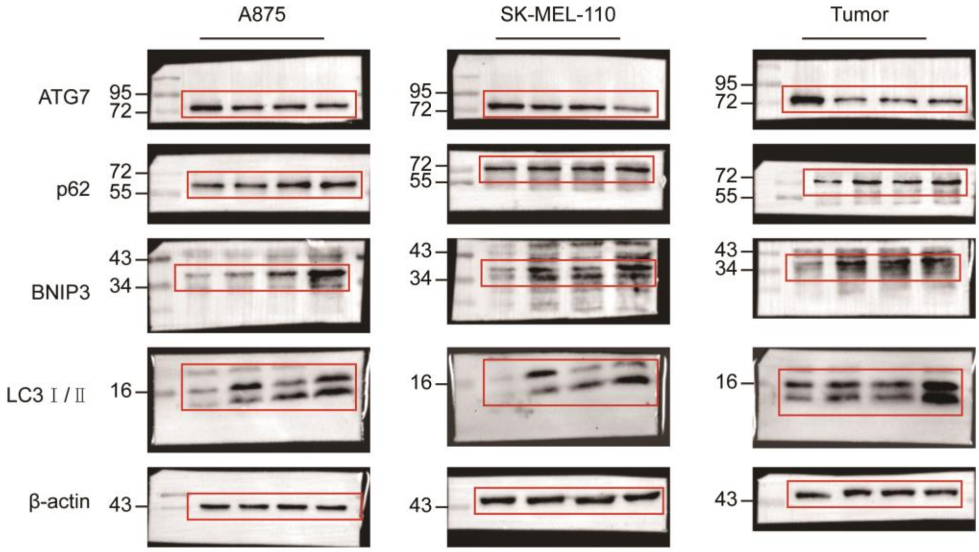

Supplement: Supplementary file 6 — Western blot [file 41419_2024_7104_MOESM6_ESM.pdf]
